# Assessing the parent–infant relationship: a two-stage, COSMIN-informed systematic review evaluating clinician-rated measures

**DOI:** 10.3389/fpsyt.2025.1426198

**Published:** 2025-04-28

**Authors:** Isabelle Shone, Lynsey Gregg, Anja Wittkowski

**Affiliations:** ^1^ Division of Psychology and Mental Health, School of Health Sciences, Faculty of Biology, Medicine and Health, The University of Manchester, Manchester, United Kingdom; ^2^ The Perinatal Mental Health and Parenting (PRIME) Research Unit, Greater Manchester Mental Health National Health Service (NHS) Foundation Trust, Manchester, United Kingdom; ^3^ Manchester Academic Health Sciences Centre, Manchester, United Kingdom

**Keywords:** reliability, validity, quality appraisal, staff, perinatal, mothers, psychometric properties

## Abstract

**Background:**

The parent–infant relationship is important for healthy infant development. Parent–infant assessments can aid clinicians in identifying any difficulties within the parent–infant relationship. Meaningful, valid, and reliable clinician-rated measures assist these assessments and provide diagnostic, prognostic, and treatment indications. Thus, this review aimed to (a) provide a comprehensive overview of existing clinician-rated measures and their clinical utility for the assessment of aspects of the parent–infant relationship and (b) evaluate their methodological qualities and psychometric properties.

**Methods:**

A systematic search of five databases was undertaken in two stages. In Stage 1, relevant clinician-rated parent–infant assessment measures, applicable from birth until 2 years postpartum were identified. In Stage 2, relevant studies describing the development and/or validation of those measures were first identified and then reviewed. Eligible studies from Stage 2 were quality assessed in terms of their methodological quality and risk of bias; a quality appraisal of each measure’s psychometric properties and an overall grading of the quality of evidence were also undertaken. The COnsensus-based Standards for the selection of health Measurement INstruments methodology was used.

**Results:**

Forty-one measures were eligible for inclusion at Stage 1, but relevant studies reporting on the development and/or validation of the parent–infant assessments were identified for 25 clinician-rated measures. Thirty-one studies reporting on those 25 measures that met inclusion criteria were synthesised at Stage 2. Most measures were rated as “low” or “very low” overall quality according to the Grading of Recommendations Assessment, Development and Evaluation approach. The most promising evidence was identified for the *Mother–Infant/Toddler Feeding Scale, Tuned-In Parenting Scale*, and *Coding Interactive Behaviour Instrument.*

**Conclusions:**

There was a notable diversity of measures that can be used to assess various aspects of the parent–infant relationship, including attunement, attachment, interaction quality, sensitivity, responsivity, and reciprocity. The quality of methodological and psychometric evidence across the reviewed measures was low, with 76% of measures having only one study supporting the measure’s development and/or validation. Thus, further research is needed to review the psychometric properties and suitability as assessment measures.

## Introduction

Disruptions to early childhood, for example, through trauma or illness, can have a long-term impact on infant mental and physical health, developmental trajectory, and even socioeconomic standing later in life (1, 2). During the first critical year in a child’s life, the infant brain undergoes rapid development and is particularly sensitive to experiences, both positive and negative (3). The parent–infant relationship has been identified as an early life experience, crucial for the infant’s development (4, 5). As infants can recognise and respond to parental speech and cues within the first three months of life (6), parental behaviours can significantly and profoundly influence infant wellbeing (7). Inappropriate parent–infant interactions and traumatic experiences in the early period of a child’s life can impact the developing brain (8, 9) and lead to increased cortisol levels, which may later increase the risk of hyperactivity, anxiety, and attachment difficulties (10, 11). Additionally, the quality of the parent–infant relationship is known to have a significant impact on the social and emotional development of the infant as well as on cognitive and academic development (5, 12, 13). Brief periods of poorly attuned parent–infant relationships are common; however, prolonged periods of inconsistent parenting and disorganisation within the dyad can lead to maladaptive outcomes for infants (4, 14).

The impact of perinatal mental health difficulties (PMHDs) on the parent–infant relationship has been acknowledged in the literature (15–17). PMHDs occur during pregnancy or in the first year following birth, affecting up to 20% of new and expectant mothers (18). PMHDs cover a wide range of conditions, including postpartum depression, anxiety and psychosis (19). If left untreated, PMHDs can have both short- and long-term impacts on the parent, child and wider family, including transgenerational effects (20). Perinatal mental health (PMH) services (including parent–infant services) can help to ameliorate these effects. PMH services assess the parent–infant relationship and identify negative and positive aspects of parent–infant interactions (21). The assessment of the parent–infant relationship and its associated aspects, such as attachment behaviours, sensitivity, responsivity, reciprocity, and attunement, can assist clinicians in providing assessment, guidance, and, importantly, interventions, with the aim of improving maternal sensitivity, the parent–child relationship and child behaviour (22). Measurement tools are also routinely used to monitor and evaluate treatment and service effectiveness. It is therefore of critical importance for clinicians to have access to meaningful, valid, and reliable measures to assess the parent–infant relationship.

The Royal College of Psychiatrists (23) recommends several parent report measures to assess the parent–infant relationship, namely, the *Postpartum Bonding Questionnaire* (PBQ) (24) and the *Mothers’ Object Relations Scale–short form* (25) as well as clinician-rated measures, such as the *Bethlem Mother–Infant Interaction Scale* (26), the *CARE-Index* (27), *the Parent*–*Infant Interaction Observation Scale* (28), and the *National Institute of Child Health and Human Development scale* (NICHD) (29).

In a comprehensive review of 17 original parent report assessment measures and 13 modified versions, Wittkowski et al. (30) identified that the PBQ, both the original and modified versions, was found to have the strongest psychometric properties with the highest quality of evidence ratings received. Despite the potential drawbacks to using clinician-rated measures, several authors [e.g., (31–33)] have questioned the benefits of parent report measures over clinician-rated or observational measures, citing possible biases from the parents regarding their child’s perceived skills, behaviours, and interactions or their tendency to respond in a socially desirable way. Wittkowski et al. (30) also wondered if clinician-rated measures might not be used consistently across services, potentially due to a need for training to use the measures and any trainingcosts, as well as supervision and capacity issues.

At least three other reviews of clinician-rated measures assessing aspects of the parent–infant relationship exist. For example, in their systematic review of 17 measures, of the parent–infant interaction, Munson and Odom (34) provided good levels of detail regarding the validity and reliability of the identified assessment measures; however, they did not assess responsiveness or measurement error and, in terms of validity, they also did not assess the measures’ structural validity, thereby reducing the comprehensiveness of their results. They also drew information from non-peer reviewed information, such as books and manuals; thus, the impact of the results in this field of research may be reduced (30). Additionally, their review, which is now 28 years old, excluded measures that used behavioural coding systems, solely assessing measures which used rating scales.

To demonstrate the appropriateness of assessing behavioural and emotional problems during infancy, Bagner et al. (35) conducted a review of both parent report questionnaires (*n* = 7) and observational coding or clinician rated procedures (*n* = 4). Of the four observational coding measures they reviewed, *the Functional Emotional Assessment Scale* (36) and the *Emotional Availability Scales* (*EAS*) (37) are the most widely known ones. The authors concluded that the observational coding procedures provided more detailed and meaningful information regarding the infant (less than 12 months old) and caregiver than parent report measures. However, their review did not assess responsiveness, measurement error, or hypothesis testing for construct validity to determine the quality of the studies, potentially leading to errors in judgement when clincians or researchers attempt to determine the best measure to use (32).

Finally, in their comprehensive review of measures rated by a trained clinician, Lotzin et al. (32) focused on 24 existing measures with more than one journal article describing or evaluating each measure. They synthesised 104 articles published between 1975 and 2012, 60.5% of which had low methodological quality. Lotzin et al. (32) assigned lower quality ratings to authors not reporting enough detail about their study and/or using small sample sizes. Lotzin et al. (32) also concluded that further studies refining the existing tools were needed with regard to content validity and consequential validity. Although they were comprehensive and thorough in their evaluation of psychometric properties across their stipulated five validity domains of (1) content, (2) response process, (3) internal structure, (4) relation to other variables and (5) consequences of assessment, Lotzin et al. (32) appeared to follow their own idiosyncratic method of assessing a measure’s validity, rather than following standardised criteria.

Increasingly, systematic reviews of assessment measures (self-report and/or clinician-rated) have used the COnsensus-based Standards for the selection of health Measurement INstruments (COSMIN) (38–41) tools. The COSMIN is an initiative of a team of researchers who have expertise in the development and evaluation of outcome measurement instruments. The COSMIN initiative aims to improve the selection of outcome measures within clinical practice and research (41) by developing specific standards and criteria for evaluating and reporting on the measurement properties of the outcome measures (42). For examples of reviews informed by the COSMIN criteria and guidelines, see Wittkowski et al. (43), Bentley, Hartley and Bucci (44) and Wittkowski et al. (30). These reviews did not assess clinician-rated measures.

Given the shortcomings of the abovementioned reviews by Wittkowski et al. (30), Munson and Odom (34), Bagner et al. (35) and Lotzin et al. (32), there is now a clear need for a systematic, transparent, comprehensive, COSMIN-informed review of relevant measures in this field. Thus, the aim of this systematic review was to assist practitioners and researchers in identifying the most suitable measures to use in their clinical practice or research by providing an overview (in Stage 1) and evaluation (Stage 2) of the current existing clinician-rated assessment measures of the parent–infant relationship, including its specific aspects such as attachment behaviours, sensitivity, responsivity, reciprocity, and attunement. The following questions were examined in this review:

1. What assessment measures did exist for clinicians to assess the parent–infant relationship in the perinatal period?
2. Which measures demonstrated the best clinical utility, methodological qualities, and psychometric properties?

## Methods

This systematic review, registered with the PROSPERO database (www.crd.york.ac.uk/prospero; registration number CRD42024501229), was conducted in accordance with the COSMIN tools (38, 41, 42) and the Preferred Reporting Items for Systematic Reviews and Meta-Analyses (PRISMA) guidelines (45). The methodology, which was specifically developed and validated for use in reviews of patient-reported outcome measures (PROMS) (38), can be adapted and used for other types of outcome measures, for example, those in which opinions on the parent–infant relationship are not self-reported but instead are evaluated by clinicians (clinician-reported outcome measures or ClinROMs) (40). The first author acted as the main reviewer but received support and supervision from the other two authors.

### Search strategy

A search was conducted in two stages: 1) to identify which parent–infant assessments exist for clinicians to use and 2) to identify studies describing the development and/or validation of each identified measure. The following databases were searched for both stages: PsycINFO (Ovid), Cumulative Index of Nursing and Allied Health Literature (CINAHL), Excerpta Medica database (EMBASE, Ovid), Medical Literature Analysis and Retrieval System Online (MEDLINE, Ovid), and Web of Science.

Stage 1 of the search involved designing a search strategy to identify and retrieve studies of relevance to the development and/or use of clinician-rated measures of the parent–infant relationship. As recommended by the COSMIN guidelines for systematic reviews (41), this initial search was first piloted and then, after further refinement with a university librarian, the final Stage 1 search was completed in November 2023. Searches using Ovid (MEDLINE, EMBASE and PsychINFO) were limited to abstracts, English language and “humans.” CINAHL and Web of Science did not offer these options of limits. Six search categories were developed, which were combined using the Boolean operator “AND.” The instruction “OR” was applied within each category and, when relevant, wildcard asterisks were used to capture related terms (**Table 1**). At Stage 1, all articles were screened based on abstract and title review and those mentioning parent–infant assessment measures were examined for full-text review. Each identified measure was assessed for eligibility against the inclusion and exclusion criteria.

**Table 1:** Search terms and limits at Stage 1.

Database (and platform): PsycInfo (OVID); Medline (OVID), EMBASE (OVID); CINAHL plus (EBSCOhost); Web of Science (Clarivate)	
1.	(Parent* or maternal or paternal or mother* or father* or caregiver* or guardian*) AND (Infant* or child* or newborn or baby or neonate or babie*)
2.	(Parent–infant) OR (infant–parent) OR (mother–infant) OR (infant–mother) OR (father–infant) OR (infant–father) OR (caregiver–infant) OR (infant–caregiver)
3.	Relations* OR interact* OR communicat* OR bond* OR attachment OR “nonverbal communicat*” OR dyadic behavio* OR “interpersonal relation*” OR “mother–child relation*” OR “father–child relation*” OR “parent–infant relation*” OR synchrony OR synchronicity OR “emotional availability” OR attitude* OR belief* OR responsiv* OR feel*
4.	Perinat* OR antenat* OR prenat* OR puerper* OR postnat* OR postpart* OR peripartum
5.	Assess* OR observation* OR behavio* OR measur* OR scale* OR tool* OR inventor* OR instrument* OR test* OR rat* OR behavio* cod* OR behavio* assessment* OR behavio* measure* OR rat* scale* OR cod* system* OR checklist* OR videotap* OR video* record*
6.	Clinician* OR staff OR practitioner* OR observer* OR rater* OR professional*
7.	1 AND 2 AND 3 AND 4 AND 5 AND 6

Limits: Abstract, Humans and English language.

To ensure the reliability of this review process, an independent reviewer (E.W.) double screened 10% of randomly selected papers from Stage 1. Cohen’s kappa and the percentage of inter-rater agreement were calculated, with good agreement (κ = .80, *p* <.001; 98.20%) (46).

At Stage 2, any relevant measures identified in Stage 1 were searched for in the same databases to identify any studies describing each measure’s initial development and/or validation. This search was conducted in December 2023 and later updated in early 2024. The following terms were searched: “Relationship” OR “Interaction” OR “Dyad” OR “Bond” OR “Sensitivity” OR “Responsiveness” OR “Attachment” OR “Attunement” OR “Reflexivity” OR “Adjustment” OR “Behaviour” AND the measure’s name OR abbreviation. Studies identified in Stage 2 were reviewed based on title and abstract; studies were assessed for eligibility by examining their full text, and their reference lists were checked for additional studies.

### Eligibility criteria of measures and studies

At Stage 1, measures were included if they were developed for clinicans to assess or rate the parent–infant relationship or a specific aspect of this relationship (e.g., attachment, reciprocity, attunement, bonding, parental sensitivity, and emotional regulation) (12). For the purpose of this review, we used the following definitions of the parent–infant relationship to help guide the identification of suitable measures: “Parent–infant relationships refer to the quality of the relationship between a baby and their parent or carer” [46, p. 2] and “the connection or bond created between the parent and infant through the exchange of behaviours and emotion communicated between both parties” [(47), p.3]. Thus, we included measures of interaction between the parent and their infant if it was a reciprocal exchange. The CARE-Index was also pre-determined to be included because its utility in assessing parent–infant interactions has been demonstrated in research into attachment behaviours (27, 48, 49). The CARE-Index has also been recognised in other systematic reviews of parent–infant assessment measures, including by Lotzin et al. (32) and the Royal College of Psychiatrists (23).

Measures were included only if they were applicable for use with an infant from birth up to the age of 2 years, which is defined as the perinatal period by the NHS Long Term Plan in the UK (50) and sometimes also referred to as the first 1,001 days (51). In the perinatal period, any difficulties within the parent–infant relationship should be identified as early as possible so that future interventions or treatment decisions can be made (52). Measures were excluded if they were designed to assess a related but different concept (e.g., “parenting style” or “attitudes to pregnancy”). Measures were also excluded if full-text studies could not be accessed or if they assessed the parent–infant relationship as part of a subscale in a longer inventory.

At Stage 2, studies were included if they 1) described the initial development and/or validation of an identified measure, 2) included data pertaining to an attempt to validate and/or to test the psychometric properties of the measure and this was stated in the aims of the study, and 3) were published in a peer reviewed journal in order to ensure consistently high-quality studies were used (53). Studies were excluded if they were not written in English and/or were reported only in theses, dissertations, or conference abstracts. We also excluded any measure for which we could not identify any studies describing the psychometric evaluation of that measure.

### Quality assessment of the studies included after Stage 2

**Figure 1:**
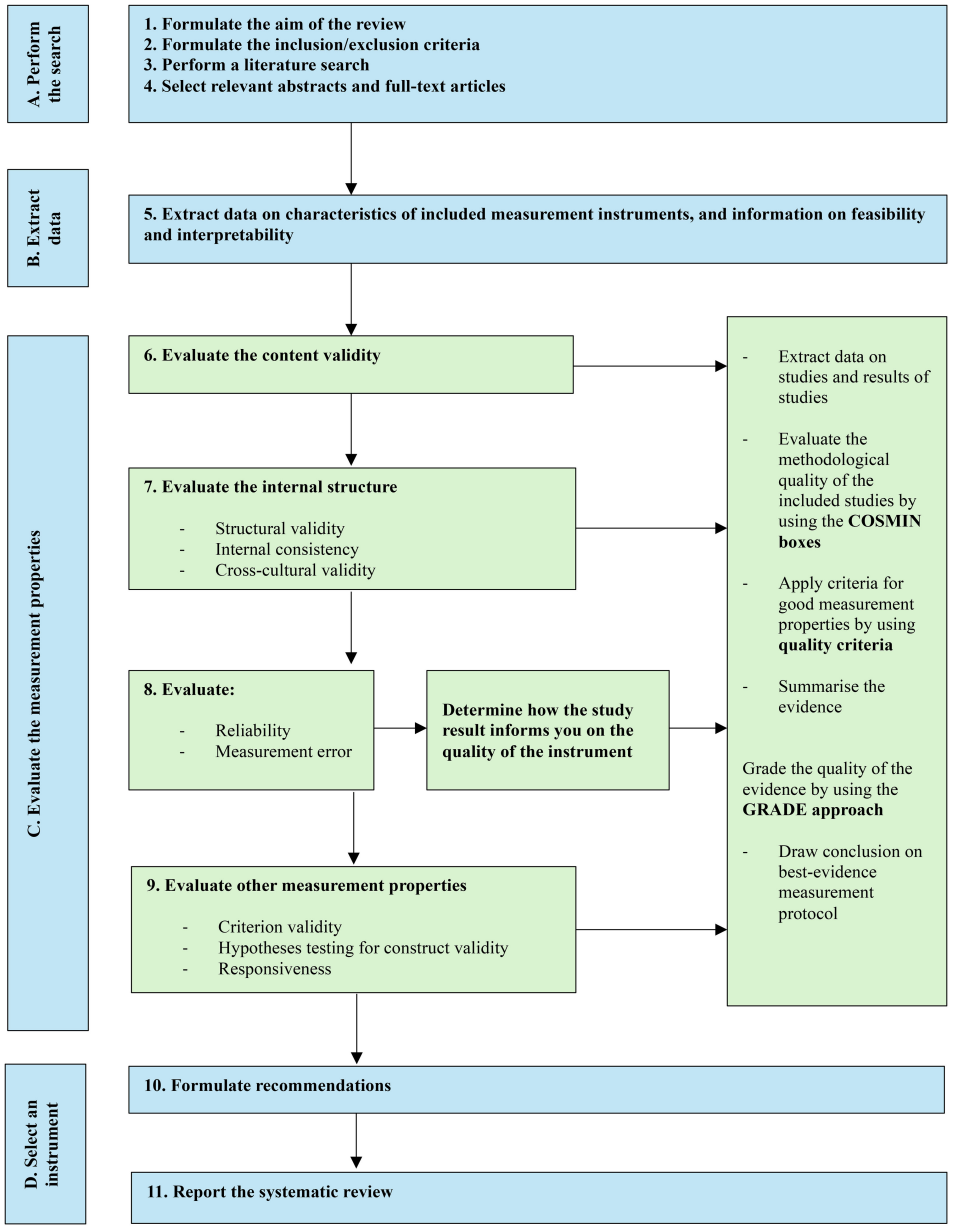
Diagram of the 11 steps for conducting a systematic review on any type of outcome measure instrument.

The COSMIN Risk of Bias Tool (40), an extended version of the COSMIN Risk of Bias checklist (38), was used to assess the methodological quality of studies identified at Stage 2, and subsequently determine each study’s overall risk of bias. **Figure 1** reflects the recommended 11-step procedure for conducting a systematic review on any type of outcome measure instrument outlined in the COSMIN Risk of Bias Tool user manual (40). The manual was developed to assess the quality of studies of all types of outcome measure instruments (including ClinROMs) and designed to be incorporated into the COSMIN methodology (40). It differs from the COSMIN Risk of Bias checklist in that it includes boxes for assessing reliability and measurement error. Furthermore, Step 8 (“Evaluate interpretability and feasibility”) was removed from the Risk of Bias Tool because interpretability and feasibility do not refer to the quality of the ClinROM (40). Interpretability and feasibility were instead extracted and summarised within a descriptive characteristics table. In this table, we included ease of administration (with regard to home or laboratory observations and time required to complete observations), associated costs and interpretability of scores. Both the COSMIN Risk of Bias Tool (40) and the COSMIN criteria (41, 42) are based on the COSMIN taxonomy for measurement properties, and these criteria are generally agreed as gold standard when evaluating measures in the context of a systematic review ensuring standardisation across papers (39). The COSMIN guidelines recommend the following stages for assessing the quality of an outcome measure, outlined in **Figure 1** as Parts A, B, and C.

### Part A: quality appraisal for the methodological quality for each measurement property and risk of bias assessment across each study

The first steps in assessing the methodological quality and risk of bias of the included studies were based on the Terwee et al. (42) COSMIN criteria and the Mokkink et al. (40) COSMIN Risk of Bias Tool. A COSMIN evaluation sheet (see **Appendix A in Supplementary Materials**) was adapted for this review to include comprehensibility (from the clinician’s point of view) because this was more applicable for clinician-rated measures (54). Content validity was assessed in terms of relevance, comprehensiveness and comprehensibility using Terwee et al.’s (42) updated criteria. Each measurement property is outlined in **Table 2**.

**Table 2:** Definitions and criteria for good measurement properties.

Measurement property	Definition	Rating	Criteria
Validity *(the degree to which the clinician-reported outcome measure (ClinROM) measures the construct(s) it purports to measure)*			
Content validity (includes relevance, comprehensiveness, and comprehensibility)	The degree to which the measure is an adequate reflection of the construct being measured.	+	Above 85% of the items of the measure are relevant AND are relevant for the target population AND are relevant for the context intended for use AND have appropriate response options OR have appropriate recall period AND include all key concepts AND together comprehensively reflect the construct intended to be measured.
		?	Not enough information for (+) OR potential biases identified OR the quality of the study is inadequate
		-	Less than 85% of the items of the measure fulfil the above criteria
Structural validity	The degree to which the scores of an assessment measure can adequately reflect the dimensionality of the construct being measured.	+	Classical Test Theory (CTT) Confirmatory factor analysis (CFA): comparative fit index (CFI) or Tucker-Lewis index (TLI) or comparable measure > 0.95 OR Root Mean Square Error or Approximation (RMSEA) < 0.06 OR Standardised Root Mean Square Residuals (SRMR) < 0.08^a^ Item Response Theory (IRT)/Rasch No violation of unidimensionality^b^: CFI or TLI or comparable measure > 0.95 OR RMSEA < 0.06 or SRMR < 0.08 AND No violation of local independence: residual correlations among the items after controlling for the dominant factor < 0.20 OR Q3s < 0.37 AND No violation of monotonicity: adequate looking graphs OR item scalability > 0.30 AND Adequate model fit IRT: *_X_* ^2^ > 0.001 Rasch: infit and outfit mean squares ≥; 0.5 and ¾ 1.5 OR Z-standardised values > - 2 and < 2
		?	CTT: not all information for (+) reported IRT/Rasch: Model fit not reported
		-	Criteria for (+) not met
Hypothesis testing for construct validity	The degree to which the scores of a measure are consistent with hypotheses based on the assumption that the outcome measure validly measures the construct being measured.	+	At least 75% of the results are in accordance with the hypothesis
		?	No hypothesis defined (by the review team)
		-	Less than 75% of the results are in accordance with the hypothesis
Criterion validity	The extent to which the scores of the measure are an adequate reflection of a “gold standard.”	+	Correlation with gold standard ≥; 0.70 OR area under the curve (AUC) ≥; 0.70
		?	Not all information for (+) reported
		-	Correlation with gold standard < 0.70 OR AUC ≥; 0.70
Reliability (the degree to which the measurement is free from measurement error)			
Internal consistency	The degree of interrelatedness among the items of the assessment measure.	+	At least low evidence (as per GRADE) for sufficient validity AND Cronbach’s alpha(s) ≥; 0.70 for each unidimensional scale or subscale
		?	Criteria for “at least low evidence (as per GRADE) for sufficient structural validity” not met
		-	At least low evidence (as per GRADE) for sufficient structural validity AND Cronbach’s alpha(s) < 0.70 for each unidimensional scale or subscale
Reliability	The scores given by clinicians are the same for repeated measurement under different conditions (i.e., test–retest, inter-rater, intra-rater).	+	Intraclass correlation coefficient (ICC), weighted Kappa, Pearson or Spearman correlation coefficient ≥; 0.70
		?	ICC, weighted kappa, Pearson or Spearman correlation coefficient not reported
		-	ICC, weighted kappa, Pearson or Spearman correlation coefficient < 0.70
Measurement error	The degree to which the systematic and random error of scores is not attributed to the changes in the construct being measured.	+	Smallest detectable change (SDC) or limits of agreement (LoA) or CV*√2*1.96 < minimal important change (MIC), % specific agreement > 80%
		?	MIC not defined
		-	SDC or LoA > MIC, % specific agreement < 80%
Responsiveness	The ability of an instrument to detect change over time within the construct being measured.	+	At least 75% of the results are in accordance with the hypothesis OR AUC ≥; 0.70
		?	No hypothesis defined (by the review team)
		-	Less than 75% of the results are in accordance with the hypothesis OR AUC < 0.70

The criteria are based on Terwee et al. (42), Prinsen et al. (41) and Mokkink et al. (40).

(+) = sufficient, (−) = insufficient, ()? = indeterminate.

a To rate the quality of the summary score, the factor structures should be equal across studies.

b Unidimensionality refers to a factor analysis per subscale, while structural validity refers to a factor analysis of a (multidimensional) outcome measure.Background colours were chosen to reflect the graded scoring system in place.

Each study was assessed for methodological quality and was rated using the COSMIN scale’s 4-point scoring system (4 = “very good,” 3 = “adequate,” 2 = “doubtful,” 1 = “inadequate”). An overall score for a study’s risk of bias was then determined by taking the lowest rating among all criteria for each category, known as the “worst score counts” method. This method was followed because poor methodological qualities should not be compensated for by good qualities (42).

### Part B: quality appraisal of the psychometric properties of each measure

The main reviewer appraised the quality of the reported results in terms of psychometric properties for each measure. Each of the eight psychometric properties (except content validity) was rated as “sufficient” (+) if results were determined to provide good evidence of a measure exhibiting this property, an “indeterminate” (?) rating was assigned if results were not consistent, not reported, or appropriate tests had not been performed and an “insufficient” (−) rating was assigned when appropriate tests had been performed, but the results were below the COSMIN checklist’s standards.

Content validity (i.e., in terms of relevance, comprehensiveness and comprehensibility) was rated as either sufficient (+), insufficient (−), inconsistent (±) or indeterminate (?). A subjective rating regarding content validity was also considered (41). The evaluated results of all studies for each measure were summarised. The focus at this stage changed to the measures, whereas in the previous substeps, the focus was on the individual studies.

### Part C: quality grading of the evidence

The strength of evidence for each category for each measure was determined based on the methodological quality and risk of bias (Part A) and the psychometric properties (Part B). The main reviewer utilised the modified Grading of Recommendations Assessment, Development and Evaluation (GRADE) approach (38) to assess the quality of the evidence provided for each measure (**Table 3**). Detailed information on the GRADE approach can be found in the COSMIN user manual (38, 41, 42). As per COSMIN guidance, if studies were rated as being “inadequate” overall (Part A), the GRADE rating of “very low” was given for the content validity categories. If studies were rated as being of “doubtful” quality overall, a GRADE rating of “low” was given for content validity categories (42). COSMIN guidelines recommend that studies determined to be “inadequate” should not be rated further. However, in order to gain a comprehensive overview of each measure, we rated all studies in full.

**Table 3:** Definitions of quality levels using the GRADE approach.

Quality level	Definition
**High**	We are very confident that the true measurement property lies close to that of the estimate of the measurement property.
**Moderate**	We are moderately confident in the measurement property estimate: the true measurement property is likely to be close to the estimate of the measurement property, but there is a possibility that it is substantially different.
**Low**	Our confidence in the measurement property estimate is limited: the true measurement property may be substantially different from the estimate of the measurement property.
**Very low**	We have very little confidence in the measurement property estimate: the true measurement property is likely to be substantially different from the estimate of the measurement property.

Background colours were chosen to reflect the graded scoring system in place.

As current COSMIN criteria do not include guidance regarding the rating of exploratory factor analysis (EFA), the criteria for assessing structural validity were adapted, comparable to Wittkowski et al. (30). EFAs were rated as “sufficient” if > 50% of the variance was explained (55) and studies using EFA could only be rated as “adequate” rather than “very good” for risk of bias.

When confirmatory factor analysis (CFA) was also reported alongside EFA, the lower quality evidence of EFA was ignored and the study was rated according to the CFA results reported. If the percentage of variance accounted for and/or model fit statistics were not reported in studies, an “indeterminate” rating was given.

The GRADE approach to rating results also takes into consideration the risk of bias, inconsistency (unexplained inconsistency of results across multiple studies), imprecision (total sample size in the studies) and indirectness (evidence from different populations than the population of interest) (40, 41). The GRADE approach follows the assumption that all evidence is of high quality to begin with. The quality of the evidence is subsequently downgraded to “moderate,” “low,” or “very low” when there is a risk of bias, unexplained inconsistencies in the results, imprecision (less than 100 or less than 50 participants) or indirect results (41).

## Results

### Review process

**Figure 2:**
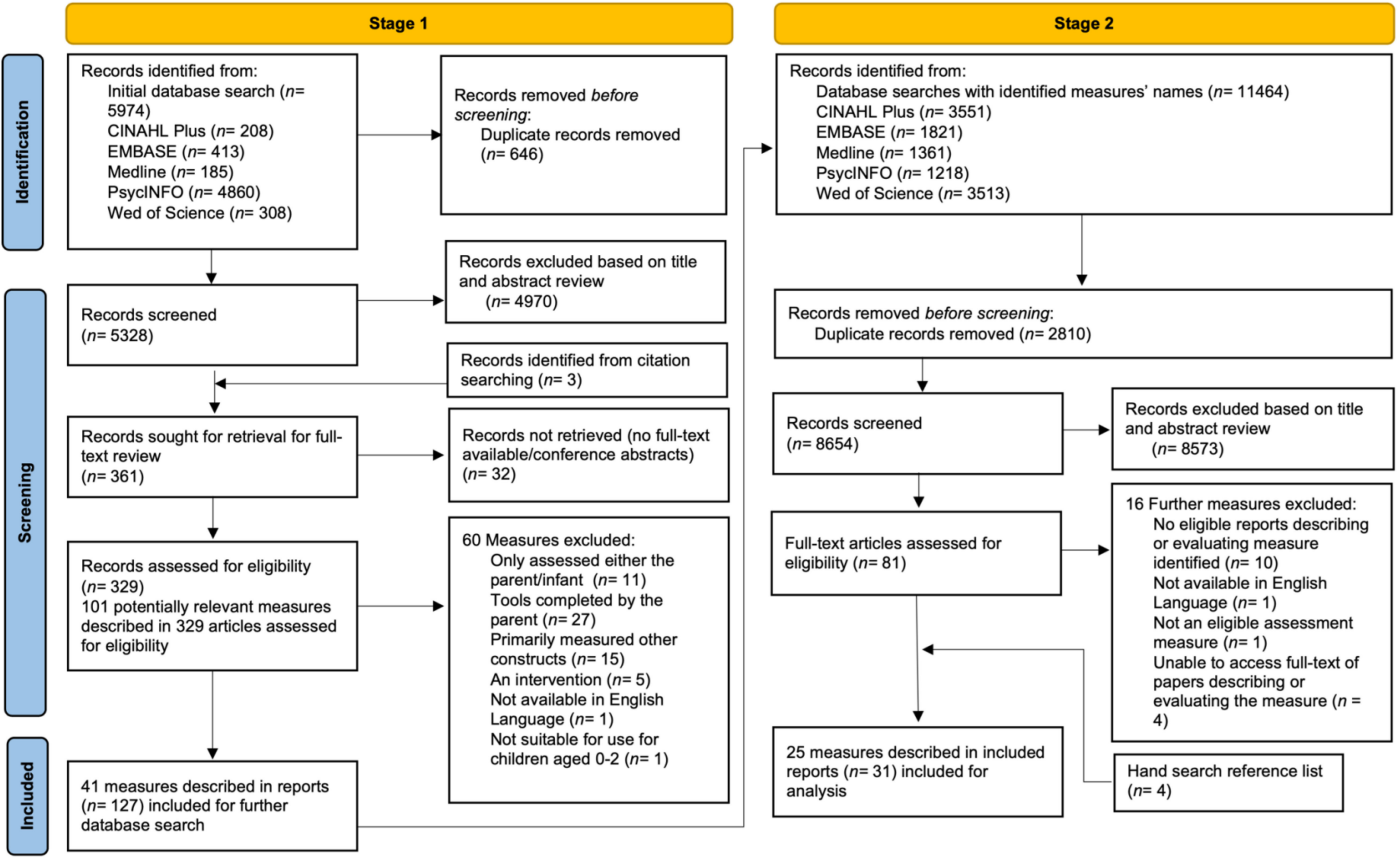
PRISMA flow diagram of both stages of the search process.

At Stage 1, 5,974 papers were identified (see **Figure 2** for details). After removing duplicates at this stage, the titles and abstracts of 5,328 records were screened. The full texts of 329 papers were examined against the inclusion and exclusion criteria, leading to the identification of 41 potentially eligible parent–infant measures.

At Stage 2, with the titles of the identified measures as the search terms, 11,464 records were identified. After removing 2,810 duplicates, 8,654 records were screened, with 8,573 records subsequently excluded based on title and abstract review. This process resulted in 81 full text articles, which were assessed for eligibility at Stage 2.

All decisions regarding inclusion and exclusion of studies and measures were discussed by all authors and any discrepancies were resolved (for a list of excluded measures, please see **Appendix B in Supplementary Materials**). After a detailed and comprehensive assessment of the identified studies from Stage 2, 31 studies describing the development of and/or validation of 25 measures were included in this review.

### Study characteristics

After completion of Stage 2, the publication dates of the included studies ranged from 1978 to 2023 and sample sizes ranged from ten (56) to 838 participants (57). The greatest number of studies came from the United States of America (USA; *n* = 19), with the remaining studies from the United Kingdom (UK; *n* = 4), Australia (*n* = 3), Denmark (*n* = 1), Germany (*n* = 1), the Netherlands (*n* = 1), Peru (*n* = 1), and Switzerland (*n* = 1). Studies were conducted using either a non-clinical sample (*n* = 17) or a clinical sample (*n* = 9), or both a clinical and non-clinical comparison sample (*n* = 5). Further details on measure development, aspects of clinical utility and characteristics of each of the included measures and studies are provided in **Table 4**.

**Table 4:** Overview of the 25 included parent–infant assessment measures (presented in alphabetical order).

	Developer(s)/author(s), date and country	Focus of measure	Scoring format			Method of assessing the parent–infant relationship and time taken to administer	Age of child assessment designed for use	Constructs assessed (parent/infant/dyad)	Development of measure including study participants, sample size and population	Details of costs and training (if applicable) and access to the measure
			Item number	Name of subscales (number of items)	Total score range/Interpretation					
1	*Assessment of Mother–Infant Sensitivity (AMIS)*									
	Price (1983) (58) USA	The quality of the early mother–infant interactions, specifically in terms of sensitivity, in a feeding context.	25	Parent scales (15 items in total): 1. Spatial distance (1) 2. Holding style (1) 3. Predominant maternal mood/affect (1) 4. Verbalisation (tone) (1) 5. Verbalisation (content) (1) 6. Visual interaction behaviour (1) 7. Modulation of distress episodes (1) 8. Caregiving style (1) 9. Stimulation of infant (1) 10. Response to changing levels of infant activity (1) 11. Burping style (1) 12. Stimulation to feed (1) 13. Manner of stimulation to feed (1) 14. Frequency of stimulation to feed (1) 15. Response to infant satiation (1) Infant scales (7 items in total): 16. Predominant infant state (1) 17. Predominant infant mood/affect (1) 18. Vocalizations (1) 19. Distress (1) 20. Visual behaviour (1) 21. Posture (1) 22. Response to stimulation to feed at satiation (1) Dyadic scales (3 items in total): 23. Synchrony in response to pleasurable affect (1) 24. Regulation of feeding at initiation (1) 25. Regulation of feeding at termination (1)	5-point rating scale (ranging from 1 to 5). 1–5 points for each item, so total score ranges from 25 to 125. Higher values indicate greater “sensitivity.” The items within the scale can be grouped into three classes of behaviour including: holding/handling, social/affective and feeding/caregiving.	Ratings are made from observations of 15- to 30-min videotaped recordings of the mother–infant interactions. Total scores are given from observations of the entire videotaped interaction rather than sections within the videotaped recording. Dyads can be observed at home or in clinic settings. Maternal behaviour is not scored within the context of the infant’s needs or the mother’s response to infant behaviour; thus, the scale is designed to identify areas of less sensitive maternal behaviour.	0–3 months. A focus on feeding, so the scale intended to evaluate breastfeeding and/or bottle feeding of a baby less than 3 months old. Thus, limited utility in other contexts and outside of this age range.	Parent, infant and dyad	The study sample consisted of 53 dyads in a feeding-context. The infants were 4–6 weeks old. Ethnicity and socioeconomic status not reported. No further information given in text on sample used or measure development (Price, 1983) (58). Non-clinical sample.	Costs: Not applicable Scale is free to access in the appendix of Price (1983) (58). Training: No specific training courses or reliability assessments required or reported online or in study (Price, 1983) to utilise this measure. Access: Scale is freely available and easily accessible in the Appendix of Price (1983) (58).
2	*Attachment Q sort*									
	Waters and Deane (1985) (59) USA	An assessment of the parent–infant relationship within the context of attachment. The Q-set covers a broad range of secure base and exploratory behaviour, affective responses, social referencing behaviours and social cognition.	100	Parent observed for the following constructs (100 items in total): 1.Attachment/exploration balance (12) 2. Differential responsiveness to parents (9) 3. Affectivity (19) 4. Social interaction (18) 5. Object manipulation (14) 6. Independence/dependency (14) 7. Social perceptiveness (8) 8. Endurance/resiliency (6) Infant observed for following constructs: Security Dependency Sociability Social desirability	Rankings range from perfectly positive score (1.0 – secure attachment) to perfectly negative score (−1.0 – insecure attachment). Clinicians are required to rank order items on cards on a Q-sort grid. Each item is scored in terms of its placement (piles 1–9) on the Q-sort grid (e.g., items in pile 9 receive scores of 9, items in pile 1 receive scores of 1). Printed on each card is each item’s title and more specific descriptive statements. These items make specific reference to a behaviour of the parent or infant and make up the Q-set.	Live observations by clinicians of the child interacting with the parent. It is recommended observers complete 6–8h of observations over 2 occasions to complete the Q sort. Dyads can be observed at home or in clinic settings.	12–48 months Not suitable for newborns	Parent and infant scales	This measure was developed and revised over several stages to subsequently compile a 100 item Q-set. This was described as developed through home visits and reviewing relevant literature (Waters & Deane, 1985) (59). Forty-three PhD experts provided Q-sort definitions of the relevant constructs regarding attachment behaviours. Over 100 infants and their mothers were recruited to develop the Q-set. The infants were aged 12–36 months. Ethnicity and socioeconomic status not reported. Non-clinical sample.	Costs: Q-set items free to access in appendix of Waters and Dean (1985) (59). Training: No specific training courses identified or available online. Stated in paper that observers need to be trained and able to observe the child for an average of 6–8h. Reliability assessments required. Access: Q-set items free to access in freely available in appendix of Waters and Dean (1985) (59).
3	*Bethlem Mother–Infant Interaction Scale (BMIS)*									
	Kumar and Hipwell (1996) (26) UK	Assessing the quality of mother–infant interactions in a psychiatric context	7	Parent scales (6 items in total): 1. Eye contact (1) 2. Physical contact (1) 3. Vocal contact (1) 4. Mother’s mood (1) 5. General routine (1) 6. Assessment of risk (1) Infant scale (1 item in total): 7. Baby’s contribution to interaction (1)	5-point rating scale (ranging from 0 to 5). 0 indicates the mother is interacting with the child in an appropriate, sensitive and well-organised way. A score of 3 indicates the mother was unable to sustain any meaningful dialogue or interaction with the infant. A score of 4 indicates very severe disturbances of maternal behaviour resulting in physical separation from the child.	A seven-day observation period. Scored by two members of staff on a weekly basis. Staff members are instructed to review their clinical notes from the past week, usually during handover meetings and arrive at a consensus as to the worst level of interaction observed over the past week. Designed and validated for use in inpatient settings.	0–12 months	Parent and infant scales	Measure developed out of clinical descriptions of key areas of disturbance and dysfunction. Study authors describe the use of pilot work to finalise subscales. Kumar and Hipwell (1996) also described developing descriptive anchor points into the scale to reduce the influence of bias from the raters and facilitate ratings. The study sample consisted of 78 mothers (age 18–41 years) and 78 infants (1–36 weeks) in a Mother and Baby Unit. Ethnicity not reported. The admission had to have lasted longer than 2 weeks so that at least two sets of nurse’s observations could be made. Clinical sample.	Costs: Scale is free to access and available online. https://www.rcpsych.ac.uk/docs/default-source/improving-care/better-mh-policy/college-reports/college-report-cr216.pdf?sfvrsn=12b1e81c_2 Training: This measure is aimed to be completed by nurses who were in daily contact with the mothers on the ward. So it is assumed a clinical training background is required; however, the study authors specified that the aim of developing the measure was to remove the need for specialist training for the raters. Access: No specific training courses or reliability assessments required or reported in study (Kumar & Hipwell, 1996) (26) to utilise this measure. Scale is free to access.
4	*Child-Adult Relationship Experimental Index (CARE-Index)*									
	Crittenden (1988) (27) USA	The quality of the adult–infant interaction. Focuses on assessing adult sensitivity in order to identify any risks	14	Parent scores (3 items in total): 1. Sensitive (1) 2. Controlling (1) 3. Unresponsive (1) Infant scores (4 items in total): 4. Cooperative (1) 5. Difficult (1) 6. Passive (1) 7. Compulsive Compliant (1)	Allocation of 14 points among three adult patterns and, separately 14 points among the four child patterns of behaviour. The scores on the scales range from 0 to 14, with 0 sensitivity being dangerously insensitive, 7 normally sensitive and 14 outstandingly sensitive.	Measure requires 3–5 min of semi structured play observation, which is then videotaped and coded by trained coders. Can be completed at home or in clinical settings.	0–48 months	Parent and infant scales (adult–infant interactions – the adult does not have to be the parent)	Description of measure development not given. Study sample consisted of 121 mother–child dyads. Families were referred via social services or the public health department. referrals of “maltreating” families, mostly of very low-income. The children ranged in age from 2 to 48 months. Mothers age range was 15–38 years. Ethnicity of infants was reported as either Caucasian or African American. No information on socioeconomic status reported. Each family was seen four times. Non-clinical sample.	Costs: Manual not free to access. Online training costs £850–£1,050. Training: Nine days, includes teaching, the manual and a reliability test. Access: training accessible through https://www.psychologyexperts.org/2018/02/20/care-index-training/
5	*Coding Interactive Behaviour (CIB)*									
	Feldman (2012) (60) (Information taken from Stuart et al., 2023) (57) Denmark	Parent–infant interaction quality, with a focus on social interactions	33	Parent scales (18 items in total): 1. Parental sensitivity 2. Parental intrusiveness 3. Parental limit setting Infant scales (8 items in total): 4. Child social involvement 5. Child withdrawal/negative emotionality Dyadic scales (5 items in total): 6. Dyadic reciprocity 7. Dyadic negative states As well as two items representing the lead-lag of the interaction.	5-point Likert scale (ranging from 1 to 5). All items are rated on a scale ranging from 1 representing a minimum level of behaviour, to 5 representing a maximum level of the attitude/behaviour. Half-point increases. Total score ranges from 33 to 165. Clinicians first examine the parent and infant behaviours separately, followed by the interactions between the dyad.	Observations of videotaped recordings of 5 min of ‘free-play’ interactions between the adult and infant during a home visit. The recording is then coded by trained coders.	2–36 months	Infant, parent and dyad	Measure described to have been developed based on theory and research in the field of early social development (Stuart et al., 2023) (57). 419 mother–infant dyads. Infants were aged 2-6 months. Clinical sample, consisting of mothers with depression and/or anxiety. Ethnicity and socioeconomic status not reported.	Costs: Manual and scale not free to access, training costs $2,500. Training: Three-day training seminar available online via Zoom. Access: Training accessible through https://ruthfeldmanlab.com/coding-schemes-interventions/
6	*Dyadic Mutuality Code (DMC)*									
	Censullo et al. (1987) (61) USA	Parent–infant interactions and levels of synchrony	6	Parent scales (2 items in total): 1. Maternal sensitive responsiveness (1) 2. Maternal pauses (1) Infant scale (1 item in total): 3. Infant clarity of cues (1) Dyadic scales (3 items in total): 4. Mutual attention (1) 5. Positive affect (1) 6. Turn-taking (1)	Each item is given score of 1 or 2 and a total score, rated as synchronous or low synchronous.). (Score of 1 or 2 for each item where 1 = absent, 2 = present) The total score ranges from 6 to 12. A score of 6–9 is ranked a low synchronous and scores of 10–12 are ranked as synchronous. The infant and adult behaviour is observed dyadically as an interactive unit.	Observations of 5 min of free-play between parent and infant, rated by clinicians with a scoring sheet. Original study described videotaping families in a laboratory setting. Unclear if can also be used by clinicians in home settings.	0–6 months	Infant, parent and dyad	The DMC evolved from the Dyadic Interaction Code The six scale items were included because it was established these items are relevant components of synchronous interaction in literature. Therefore, they represent the concept and definition of synchrony. (Censullo et al., 1987) (61). The participants were selected from a child development unit at a children’s hospital. The sample consisted of 20 term and 20 preterm infants and their mothers. Ethnicity reported as all Caucasian participants and all were described to be of comparable socioeconomic status. Clinical sample.	Costs: Unknown. Training: Study author described training a research associate to use the DMC – so there is an implication that some training is required. Access: No access to training or manual could be located online to use this measure.
7	*Emotional Availability Scales (EAS)*									
	Biringen, Robinson and Emde (2000) (37) (Information taken from: Aran et al., 2022) (62) USA	An evaluation of the quality of relationship between parent and child, in terms of levels of synchrony and sensitivity	42	Parent scales (28 items in total): 1. Sensitivity (7) 2. Structuring (7) 3. Non-intrusiveness (7) 4. Non-hostility (7) Infant scales (14 items in total): 5. Responsiveness to adult (7) 6. Involvement to adult (7)	7-point rating scale (ranging from 1 to 7). Higher values indicate higher sensitivity between the parent and infant and reflect the better overall quality of the relationship. Total score ranges from 7 to 42. Within the context of the parent–infant relationship, the observer is instructed to utilise context and clinical judgements to infer the appropriateness of observed behaviours.	Observations of the dyad at home, childcare centre, free play or structured teaching. Observers rate these interactions using a coding system. Live or videotaped. 15–20 min observation minimum.	0–14 years	Parent and Infant only (no dyadic scales)	This measure was developed for use among populations with clinical or developmental risk factors, including between depressed and nondepressed mothers and their infants. Informed by attachment research (Aran et al., 2022) (62). Study sample consisted of 35 mother–child dyads. No information on ages, socioeconomic status or ethnicity reported. Clinical sample.	Costs: Training, scale and manual costs available upon request through website. Training: 3-day face-to-face, group training or self-paced distance training using reading, lecture and practice on 10 training videos required. Training and reliability certifications required. Access: training accessible through https://emotionalavailability.com/courses/ea-basic/
8	*Family Alliance Assessment Scales for Diaper Change Play (FAAS-DCP)*									
	Rime et al. (2018) (63) Switzerland	An evaluation of the quality of family relations and family functioning	9	Parent(s)-infant ratings (9 items in total): 1. Readiness to interact (1) 2. Gaze orientation (1) 3. Inclusion of partners (1) 4. Coparental coordination (1) 5. Role organisation (1) 6. Parental scaffolding (1) 7. Shared and co-constructed activities (1) 8. Sensitivity (1) 9. Family warmth (1)	5-point rating scale (ranging from 1 to 5). The measure consists of 9 interactive dimensions, rated on a 5-point scale. A score of 5 indicates optimal functioning and a score of 1 indicates significant dysfunction is observed. Total score ranges from 9 to 45. The observation is made up of four parts, a score is thus assigned to each of these parts. The score on every dimension is then determined by summing the score on each part, ranging from 4 to 20. The observation also involves an assessment of the perceived quality of family alliance in terms of cooperative, collusive and disordered alliance.	The FAAS-DCP is structured in four parts of observations of interactions during the changing of diapers. Part one, one parent begins to change the diaper of the infant and the other observes the interaction without intervening. These roles then swap in the second part, the second parent becomes active and finishes the process of diaper changing. Both parents are together with the infant to share a moment, in the third part. Examples of this moment include, stroking, smiling at or soothing the infant. The final part involves asking the parent to have a discussion in the presence of the baby. Time taken to administer unclear. All interactions during the diaper change are recorded so that videotaped recordings can be coded according to the manual. Clinic settings only. Equipment required includes table, changing mat, diapers, a bin and four cameras.	The first three weeks of the child’s life	Dyadic/triadic interactions	The measure was developed based off Lausanne Trilogue Play (LTP, Gatta et al., 2016) (64). Whereas the LTP is based on a play task, the DCP is mainly based on a caregiving task. The difference being that a play activity was changed to a practical, caring activity mimicking everyday life. Good level of detail given in paper as to theoretical background and decision making around measure development. The coding system FAAS-DCP is modelled on the FAAS (Favez et al., 2011) (65). The measure was developed based off three validation studies, which involved one sample from two maternity wards in Switzerland. All the families were White European. The sample consisted of 44 triads and their newborns. Clinical sample.	Costs: Unknown Training: All interactions are coded so some level of training required to code. Access: FAAS-DCP coding system unpublished and not available in Rime et al. (2018) (63)or online. No training courses or access to manual could be located online to use this measure.
9	*Infant–Parent Social Interaction Code (IPSIC)*									
	Baird et al. (1992) (66) USA	Assessing the quality of the parent–infant relationship within free-play scenarios between infant and parent.	9	Parent scales (4 items in total): 1. Response contingency (1) 2. Directiveness (1) 3. Intrusiveness (1) 4. Facilitation (1) Infant scales (4 items in total): 5. Initiation (1) 6. Participation (1) 7. Signal clarity (1) 8. Intentional communicative acts (1) Dyad scales (1 item in total): 9. Theme continuity (1)	3-point rating scale (ranging from high, middle, low) A “High” score gives an indication of a higher frequency of behaviours and “low” scores indicate no specified behaviours were demonstrated. Only when social interaction occurs, can infant participation, infant initiation and dyadic theme continuity then be coded as present. Additonally, intrusiveness and facilitation are not compatible interactional constructs and so cannot co-occur.	Observations of the parent and infants’ ‘free play’, ideally at home, videoed and coded by trained coders. More than 5 min of videotaped recordings of infant–parent play is required. Coding is split into twenty fifteen-second intervals of specific behaviours. The first 5 min are not coded. Parents are asked to play as they normally do. The manual provides examples and nonexamples for each construct, additionally, five standard videotapes were developed to facilitate and aid coders in their training.	0–36 months.	Infant, parent and dyad.	A description of measure development is given as based on theoretical and empirical evidence for promoting optimal infant development. Definitions, examples, possible rationales for inclusion and references for each construct are given in Baird et al. (1992) (66). The study sample consisted of 159 infants ranging from birth to 31 months and their mothers (biological, adoptive and foster mothers). Infants were recruited from hospitals, early intervention programs and advertisements in newspapers. Infants were either normally developing, or there were identified environmental or biological risks for developmental delay. Ethnicity of the mothers was White and African-American and socioeconomic status was described as diverse. Non-clinical sample.	Costs: Coding sheet to free to access in Baird et al. (1992) (66) Training costs unknown. Training: Training required – Baird et al. (1992) also details procedures for guiding coding decisions to ensure reliability, as well as, decision trees to provide guidelines for sequencing the series of decisions required in coding. The paper also explains five 1h group training sessions and independent coding are required. Observers are required to obtain minimum of 75% exact agreement on training tapes. Access: Procedure for training coders, establishing and maintaining reliability, decision trees, standard tapes and guidelines (manual) are stated as available from the first author upon request.
10	*LoTTS Parent*–*Infant Interaction Coding Scale (LPICS)*									
	Beatty et al. (2011) (67) USA	Observational measure of the quality of parent–infant interactions. A particular focus on responsivity, sensitivity and warmth.	13	Global ratings (3 items in total): 1. Responsiveness (1) 2. Sensitivity (1) 3. Warmth (1) Behavioural counts (10 items in total): 4. Look (1) 5. Chasing (1) 6. Touch (1) 7. Negative touch (1) 8. Talk (1) 9. Negative talk (1) 10. Smile (1) 11. Grimace/frown (1) 12. Positive child response (1) 13. Negative child response (1)	The three global ratings: responsiveness, sensitivity and warmth are rated on a 3-point Likert scale (ranging from 1 to 3). The behavioural counts are noted and coded for presence of or absence of at each occurrence during the interaction. Total score ranges from 13 to 19.	The measure consists of a 4-min videotaped recording of the parent and infant playing and interacting with a toy. Either at home or clinical settings.	0–3 months	Dyad	Measure was developed based off adaptation from the Parent Infant Interaction Observation Worksheet (Beatty et al., 2011) (67), which grew out of Applied Behavioural Analysis literature. The LPICS is closely based on the Motivational Interviewing Treatment Integrity Scale in structure (Moyers et al., 2005) (68). 45 mother–infant dyads. Participants were all women who were recruited while pregnant from OB/GYN clinics in a large urban area. The measure was designed to be used with at-risk parenting populations, but not those with serious mental health disturbance. No information reported on ethnicity of the sample and all participants described as being of low socioeconomic status. Clinical and non-clinical sample.	Costs: Unknown. Training: Reliability assessment described as three rounds of coding and 8.5h of training in order to obtain adequate reliability. Access: No training courses or access to manual could be located online to use this measure.
11	*Manchester Assessment of Caregiver–Infant Interaction (MACI)*									
	Wan et al. (2017) (69) UK	Designed to encapsulate the qualities of parent, infant and dyadic interactions.	7	Caregiver (2 items in total): 1. Sensitive responsiveness (SR) (1) 2. Nondirectiveness (1) Infant (3 items in total): 3. Attentiveness to caregiver (1) 4. Affect (1) 5. Liveliness (1) Dyad (2 items in total): 6. Mutuality (1) 7. Engagement intensity (1)	7-point rating scale (ranging from 1 to 7) A sore of 1 indicates minimal/very low quality of interaction to a score of 7 indicating high incidence of the behaviour. Total score ranges from 7 to 49. The measure is made up of seven rating scales, covering broad aspects of interaction between a caregiver and their infant.	A 6- to 20-min videotaped recording of continuous play interactions. The videotaped recording starts once the dyad is settled in interaction yet the situation. The dyad are instructed to sit on the floor/mat either during a home visit or clinic premises. The parents are then instructed to engage in play as they normally would at home. Each videotaped recording is typically reviewed twice (or more), in order to note the observational sequence and initial ratings with the manual and then reviewed again so as to finalise the ratings.	3–15 months	Parent, infant and dyad	Measure described as developed in alignment with the transactional model of development (Sameroff, 2009) (70) [which parent–infant interactions at the level of genes, as bidirectional processes. It’s constructs and scale distributions are designed to be suitable for high and low risk populations (Wan et al., 2017) (69). Study sample consisted of 147 healthy parent–infant dyads at 3–10 months post-partum. Three community-based samples used. No information on ethnicity of the participants or socioeconomic status. Non-clinical sample.	Costs: Unknown. Training: 3 day face-to-face workshop. Consisting of an extensive practice phase with supervision and feedback and a two-part reliability assessment process. In total, 75–85h is spent in training and coding to achieve certification (Wan et al., 2017) (69). Access: Comprehensive coding manual and training package detailed as available in Wan et al. (2017) (69); however, no specific training courses, scale or manual could be located online or in Wan et al. (69) to use this measure.
12	*Massie-Campbell Scale of Mother–Infant Attachment Indicators During Stress (M-C ADS)*									
	Massie and Campbell (1986) 76] (71) (Information taken from Nóblega et al., 2019; Cárcamo et al., 2014) (72, 73) Peru/Netherlands	Designed to assess the quality of interactions between mothers and children, in terms of attachment and in situations of moderate stress.	14	Parent scales (7 items in total): 1. Gazing (1) 2. Vocalising (1) 3. Touching (1) 4. Response to touch (1) 5. Holding (1) 6. Affect (1) 7. Proximity (1) Infant scales (7 items in total): 8. Gazing (1) 9. Vocalising (1) 10. Touching (1) 11. Response to touch (1) 12. Holding (1) 13. Affect (1) 14. Proximity (1)	5-point rating scale (ranging from 1 to 5). (1 = very avoidant behaviour, 3 and 4 = typical attachment behaviour, 5 = clinging and unusually strong reaction to stress). Each of the behaviours is scored on a scale of 1, 2, 3, 4, 5 or as not observed. Each behaviour is then rated as either secure or insecure. A behaviour with a score of 3 or 4 is assigned a rating of secure and behaviours with scores of 1, 2, or 5 are rated as insecure. The dyad is rated as secure when < 50% of the behaviours are rated as secure; if not, they are rated as *insecure*.	Observations of the parent and infant interacting together during a mildly stressful event, lasting approximately 10 min. Examples of mildly stressful situations are given as dressing, bathing, playing, family mealtimes and mother–infant separations and reunions at a daycare centre. Videotaped recordings are scored by trained professionals.	0–18 months	Parent and Infant only (not dyad)	The developers of the ADS designed this scale to be quick and inexpensive to detect difficulties within mother–infant interactions. Another aim of measure development was to increase clinicians’ awareness of infants’ psychological development. Both mother and infant behaviour are scored. Therefore, observers separately code mother and infant attachment behaviours (Nóblega et al., 2019; Cárcamo et al., 2014) (72, 73). The study samples consisted of 32 mothers and their children all from Peru, the infants were aged between 8 and 10 months. No details regarding ethnicity or socioeconomic status reported (Nóblega et al., 2019) (72) and 69 low socioeconomic status Dutch dyads of White origin (Cárcamo et al., 2014) (73) Both non-clinical samples.	Costs: Both the manual and scale are free to access. Training: requires self-study of the manual. Training required to code interactions, as described in Nóblega et al. (2019) (72) and rating of 16 training videos to ensure reliability. Access: Both the manual and scale are free to access and can be found through the following website: https://www.allianceaimh.org/ads-scales
13	*Monadic Phases*									
	Tronick, Als and Brazelton (1980) (56) USA	A system for describing infant-adult face to face interactions. The importance of interaction (including between parents and infants) is emphasised and described as a structured system of mutually and reciprocally regulated units of behaviour.	100	Maternal scales (57 items in total): 1. Vocalisation (9) 2. Direction of Gaze (4) 3. Head orientation (13) 4. Facial Expression (13) 5. Body Position (10) 6. Specific Handling of the Infant (8) Infant scales (43 items in total): 7. Vocalisation (8) 8. Direction of Gaze (4) 9. Head orientation (9) 10. Facial Expression (13) 11. Body Position (9)	Scale consists of 100 items, each scored for presence of or absence of. Total score ranges from 0 to 100. Infant monadic phases: protest, avert, monitor, set, play and talk. Maternal monadic phases: avoid, avert, monitor, elicit, set, play, and talk.	Observations of an interaction between an infant and a parent. The mother is instructed to play with her baby. 3-min videotaped recording. Each behaviour is then categorised into monadic phases on a second-by-second basis, then analysed by observers. Videotaping is carried out in a laboratory setting. Scoring is then completed from the videotaped recordings. Each second-by-second combination of behaviours is transformed into one of seven Monadic Phases.	0-6 months	Parent and Infant only (not dyad)	Measure development described as informed by previous research on behaviour (Brazelton et al., 1975) (74) and face to face interactions (Stern et al., 1977) (75). The measure was designed to build on previous research and overcome their limitations. It is designed to segment the interaction into separate units of behaviour that are called monadic phases (Tronick, Als & Brazelton, 1980) (56). The study sample consisted of 5 infant-mother pairs. The infants age range was 80 to 92 days old. No information on ethnicity or socioeconomic status reported. Non-clinical sample.	Costs: Scoring tables can be found free in Tronick Als and Brazelton (1980) (56). Training: Specific training courses unknown. Reliability assessments required at 90% absolute agreement for each category. Access: No specific training courses or access to manual could be located online to use this measure.
14	*Mother-Infant/Toddler Feeding Scale (M-I/TFS)*									
	Chatoor et al. (1997) (76) USA	Assesses the quality of the mother–infant relationship in a feeding context	46	Parent(s)-infant ratings (46 items in total): 1. Dyadic reciprocity (16) 2. Dyadic conflict (12) 3. Talk and distraction (4) 4. Struggle for control (7) 5. Maternal non-contingency (7)	4-point Likert scale (ranging from 0 to 3). Rated on how often and how intensely each of the behaviours occurs (0 = none, 1 = a little, 2 = pretty much and 3 = very much). Total score ranges from 0 to 138. The scale consists of 46 mother and infant behaviours, which produce 5 subscale scores and are rated along 4 points at the end of the feed.	A 20-min observation completed in a research or clinical setting. Videotaped recordings of parent–infant interactions as the mother feeds the infant, rated by a trained observer. Scoring is then completed from the videotaped recordings.	1–36 months. However, it should also be noted the scale has not yet been validated for use with infants less than 5 weeks of age.	Dyad only.	Measure development described as informed by a series of studies conducted. Initially, a group of items describing relevant behaviours of mothers and infants was pooled and generated by experienced clinicians (Chatoor et al., 1997) (76). A pilot study was completed in which the study sample consisted of 20 mother–infant pairs. Infants age range: 5 weeks to 32 months. A second larger validation study was then completed with 74 infants with feeding disorders and 50 normal comparison subjects. These infants ranged from 6 weeks to 36 months. Ethnicity (White/African-American) and socioeconomic status reported. Forty infants participated in a reliability study. Infants were aged 7 months to 3 years. Ethnicity reported as White, African-American, Hispanic and Asian. Clinical and non-clinical samples.	Costs: Unknown. Training: involves scoring 10–12 feeding videotapes, comparing scores with a trained rater and achieving a reliability assessment of 80% agreement. Access: No specific training courses or access to manual could be located online to use this measure.
15	*Mutual Regulation Scales (Face-to-face still face paradigm) – MRS*									
	Tronick et al. (1978) (77) USA	Infant facial expression in response to caregiver changes in facial expressions	111	Parent scales (57 items in total): 1. Vocalising (9) 2. Head position (13) 3. Body position (10) 4. Specific handling of the infant (8) 5. Direction of gaze (4) 6. Facial expression (13) Infant scales (54 items in total): 7. Vocalisation (7) 8. Direction of gaze (4) 9. Head orientation (9) 10. Head position (2) 11. Facial expression (13) 12. Amount of movement (8) 13. Blinks (2) 14. Specific hand movements (5) 15. Specific foot movements (2) 16. Tongue placement (2)	Each item is rated “yes or “no” for presence of or absence of behaviours demonstrated. Separate ratings for parent and infant. From the videotaped recording, raters categorise and score the infant’s vocalizations, direction of gaze, head and body position, facial ex- pression and movement; and the mother’s vocalizations, head position, body position, direction of gaze, facial expression and handling of the infant.	Observations take place in a double room with a unidirectional mirror and recording system. A laboratory setting. Videotaped recordings are micro-analytically coded. The mother first interacts normally with infant for 3 min, a 30-s break, followed by remaining “still faced” for 3 min. Scoring is completed by two observers for each 1-second time interval as the tape runs at 1/7th of its normal speed.	0–10 months	Parent and infant (no dyadic scales)	Measure development was informed by previous research and studies of face-to-face interactions between mothers and infants (Tronick et al., 1978; Brazelton et al., 1975) (74, 77). Study sample consisted of 7 mothers and their healthy full-term infants, ranging in age from 1 to 4 months. Ethnicity and socioeconomic status not reported. Non-clinical sample.	Costs: Scoring sheet freely available in appendix of Tronick et al. (1978) (78). Training: Training requirements unknown. Inter-scorer reliability should be maintained at above.85 for each category scored. Access: No specific training courses or access to manual could be located online to use this measure.
16	*Mutually Responsive Orientation (MRO)*									
	Aksan, Kochanska and Ortmann. (2006) (78) USA	Mutually responsive orientation (MRO). MRO is a positive, responsive, mutually binding, and cooperative interaction between the parent and the infant	17	Parent(s)-infant ratings (17 items in total): 1. Coordinated routines (2) 2. Harmonious communication (4) 3. Mutual cooperation (5) 4. Emotional ambiance (6)	5-point rating scale (ranging from 1 to 5). With higher values indicating higher MRO. Total score ranges from 17 to 85. The study authors proposed four basic components of MRO in parent–child dyads: coordinated routines, harmonious communication, mutual cooperation, and emotional ambiance.	Home sessions or laboratory sessions, lasting 1.5–2h. Videotaped recordings are coded by trained coders. The tasks include: leisurely chore-oriented and care-giving activities, such as preparing and having a snack with the baby, free play, playing with toys, bathing and dressing the child, opening a gift together.	7–15 months	Dyad only	Measure development was informed by previous research on MRO of the parent and infant as individuals (Kochanska, 1997) (79), the current measure was designed with the aim of designing codes that explicitly captured the quality of the parent–child interaction at the dyadic level (Aksan, Kochanska & Ortmann, 2006) (78). Study sample consisted of 102 two-parent families with normally developing infants at 7 months and then 15 months old. Socioeconomic status and ethnicity reported (White Hispanic, African American, Asian Pacific Islander or other). Non-clinical sample.	Costs: Unknown. Training: No training requirements detailed in Aksan, Kochanska and Ortmann (2006) (78) and no training courses available online. Access: manual, training courses or scale could not be located online or in Aksan, Kochanska and Ortmann (2006) (78) to use this measure.
17	*Nursing Child Assessment Teaching Scales (NCATS)*									
	Barnard (1978) (80) (Information taken from Byrne &Keefe, 2003; Gross et al., 1993) (81, 82) USA	Assesses the quality of the parent–child relationship, with a focus on parent–child reciprocity and mutual adaptation.	73	Parent scales (50 items in total): 1. Sensitivity to cues (11) 2. Response to the child’s distress (11) 3. Social-emotional growth fostering (11) 4. Cognitive growth fostering behaviour (17) Infant scales (23 items in total): 5. Clarity of cues (10) 6. Responsiveness to the mother (13)	Each item is rated “yes or “no” for presence of or absence of behaviours demonstrated. (Yes answers receive a value of 1, and No answers are scored 0). Scores are summed for the six subscales to give a total score based on all items. Total score ranges from 0 to 73.	Suitable for both home and laboratory settings. Procedure lasts 30–45 min, whilst the parent and infant complete two structured tasks, during which the parent teaches the infant. Can be completed live or videotaped recordings scored later.	0–36 months	Parent and infant only	Measure development is based on literature in the areas of attachment, psychobiology and developmental psychology. The NCATS was developed through research within the Nursing Child Assessment Project. The NCATS is used during observation of the parent introducing a new skill that the infant has yet to demonstrate but is developmentally ready for (Gross et al., 1993) (82). The study sample consisted of 128 mothers and their 24- to 36-month-old children, mothers all had a clinical diagnosis of depression, socioeconomic status and ethnicity reported: African-American, Hispanic and Asian women (Gross et al., 1993) (82). As well as, a sample of 171 parent–child dyads, Hispanic ethnicity of low income backgrounds, the infants ranged in age from 5 to 36 months (Byrne and Keefe, 2003) (81). Clinical and non-clinical samples.	Costs: Unknown. Training: A structured 2.5-day training course is offered by certified instructors. Each learner must purchase the NCATS manual and pass a reliability test at 0.85 for clinical use or 0.90 for research use. Access: This training is outlined in Byrne and Keefe (2003) (81); however, access to training courses, scale and manual could not be located online.
18	*Parent*–*Child Early Relational Assessment (PCERA)*									
	Clark (1999) (83) USA	Assesses quality of affect and behaviour, or tone of the parent–infant relationship.	65	Parent scales (29 items in total): 1. Positive affective involvement and verbalisation (*) 2. Negative affect and behaviour 3. Intrusiveness, insensitivity, inconsistency Infant scales (28 items in total): 4. Positive affect, communicative and social skills 5. Quality of play, interest and attentional skills 6. Dysregulation and irritability Dyad scales (8 items in total): 7. Mutuality and reciprocity 8. Disorganisation and tension *exact distribution of items within subscales unknown.	5-point Likert scale (ranging from 1 to 5). 1–2 = area of concern, 3 = area of some concern, 4–5 = area of strength. Total score ranging from 65 to 325. All items are rated so that high scores indicate more positive parent–infant interactions. Raters focus on rating 8 to 10 of the variables at a time for each pass through (a total of seven to nine viewings of the entire 5-min videotaped interactions).	Measure consists of 5 min of parent and infant interactions, including a structured task, a feeding observation and free play. Videotaped. Designed to be completed in both clinical and research settings.	0–5 years	Infant, parent and dyad	Measure was originally developed for use with psychiatrically ill mothers and their infants. Developed to assess and describe the patterns of interaction and to focus early intervention efforts at improving parenting skills and the quality of the relationship (Clark, 1999) (83). Now designed to assess interaction quality in mothers with and without a history of psychiatric disorders. Sample size consisted of 359 mothers and their infants aged 10–14 months. Socioeconomic status (household income and education) reported. Ethnicity reported as White, African-American, Native American and Asian American. Clinical and non-clinical sample.	Costs: Unknown. Training: Unknown. Access: manual, training courses, or scale could not be located online to use this measure or reported in paper (Clark, 1999) (83).
19	*Parent Infant Interaction Observation Scale (PIIOS)*									
	Svanberg, Barlow and Tigbe (2013) (50) UK	Parental sensitivity and responsiveness, with the aim to identify families at high risk of parent–infant interaction problems	16	Interactional constructs of scale (16 items in total): 1. Eye contact and face-to-face placement (1) 2. Vocalisations (1) 3. Affective engagement and synchrony (1) 4. Warmth and mutual affection (1) 5. Holding and handling (1) 6. Verbal commenting about baby (1) 7. Attunement to distress (1) 8. Bodily intrusiveness (1) 9. Age appropriateness of chosen activity (1) 10. Expressed expectations about the baby (1) 11. Mind-mindedness (1) 12. Empathic understanding (1) 13. Responsive turn taking (1) 14. Gaze (1) 15. ‘Looming in’ (1) 16. Baby’s self‐soothing strategies (1)	3-point Likert scale (ranging from 0 to 4). (0 = sensitively responsive/no concern, 2 = some problems or 4 = extensive problems/considerable concern). Total score ranging from 0 to 64. The three interactional patterns of behaviour: - Sensitive responsivity. - Intrusive and over-engaged. - Unresponsive un-engaged.	Recorded observation of the parent–infant interaction over a 3- to 4-min period. Suitable for home, clinical or laboratory conditions. Videotaped recordings are then analysed by trained coders.	6 weeks -7 months Time limited infant age range of only around a 5-month period.	Dyad	The PIIOS was designed to be implemented as part of the English Healthy Child Programme and includes a number of constructs based on the CARE-Index (Crittenden, 1988) (27) so the measure is informed by attachment research. The measure also contains constructs based on research around the importance of ‘mind-mindedness’ (Meins et al., 2003) (84) in which the parent exhibits an ability to interpret and verbalise the infant’s thoughts or motivations, this has also been suggested to predict infant attachment security (Meins et al., 2001) (85). Measure was designed to identify families at low, medium and high risk of parent–infant interaction difficulties (Svanberg, Barlow and Tigbe, 2013) (50). Study sample consisted of 14 videotaped recordings of parent–infant dyads. No details on socioeconomic status, ethnicity of infant ages given. Non-clinical sample.	Costs: Scale not free to access. Training/manual/scale costs £450. Training: Online or in-person 3-day training course, plus access to manual/scale and supervision. Access: Training, the manual and scale can be accessed through the following website: https://warwick.ac.uk/fac/sci/med/study/cpd/cpd/piios/#:~:text=PIIOS%20training%20is%20usually%20delivered,intrusive%20and%20over%2Dengaged
20	*Parent*–*Infant Interaction Scale (PIIS)*									
	Clark and Seifer (1985) (28) USA	Interaction style in parent–infant dyads, including parental sensitivity and reciprocity of their interactions.	10	Parent scales (7 items in total): 1. Acknowledging (1) 2. Imitating (1) 3. Expanding/Elaborating (1) 4. Parent direction of gaze (1) 5. Parent affect (1) 6. Forcing (1) 7. Overriding (1) Infant scales (2 items in total): 8. Child social referencing (1) 9. Child gaze aversion (1) Dyadic scales (1 item in total): 10. Dyadic reciprocity (1)	5-point rating scale (ranging from 1 to 5). The scores range from 1 (poor), 3 (moderate) and 5 (excellent quality of parent–child interaction). Points 2 and 4 are used as intermediate scores. Total score ranges from 10 to 50. The 10 scales fall into three categories: Interaction style, social referencing and assessment of context.	Mothers are instructed to play with their baby as they normally would at home. The families are observed and videorecorded for 8 min. The videotaped recordings of parent–infant interactions are coded by raters. Designed to be completed in clinical or research environments, not at home.	0-18 months	Infant, parent and dyad	The measure was developed to assess interaction style in parents–infants relationships when the infant is developmentally delayed. Developed based on previous research around infant communication, parental responsiveness and parental sensitivity (Clark & Seifer, 1985) (28). This measure was developed to assess parental sensitivity to infant behaviour and reciprocity in an unstructured play session. Study sample consisted of six mothers and their infants. The infants were high risk and/or neurologically impaired. Clinical sample.	Costs: Unknown. Training: Unknown. Access: Manual, training courses or scale could not be located online to use this measure or reported in Clark and Seifer (1985) (28).
21	*Parent*–*Infant Observation Guide (PIOG) (also written in literature as the Parent*–*Child Observation Guide)*									
	Hans, Bernstein and Percansky (1991) (86) USA	Focus on the quality of the parent–infant relationship, in order to identify strengths and possible concerns in the relationship.	33	Parent scales (22 items in total): 1. Parent sensitivity to child (11) 2. Parent teaching child (7) 3. Parent effective discipline (4) Infant scales (11 items in total): 5. Child positive involvement with parent (9) 6. Child noncompliant behaviour (2)	2-point rating scale (ranging from 0 to 1). All items are rated as either observed or not observed. (Observed items are scored 1, items not observed are scored 0) Total score ranges from 0 to 33. Focus on one member of the dyad at one time, but always in relation/context of the other member of the dyad.	Observations of feeding, play and caregiving behaviours (such as changing diapers). 10 min required to complete. Observations can be completed in home, clinic or laboratory settings. Live, video recordings not required.	4–15 months	Parent and infant only	Collaboration of the measure authors, community practitioners and mothers within the community, over two years, led to the measure development. This measure was developed based on previous research and psychological theory around working with families (Hans, Bernstein & Percansky, 1991) (86). Four samples of high-risk mothers and infants: 1. 82 low-income mothers (African-American) and their 12 month old infants. 2. 42 low-income (African-American) mothers and their 6-month old infants. 3. 48 low-income (African-American) mothers and their 12 month old infants. 4. 51 adolescent parents from a variety of racial and ethnic groups and their infants between 4 and 15 months. Non-clinical sample.	Costs: Unknown. Training: Unknown, potentially not required as reported in Lotzin et al. (2015) (32) who indicated that practitioners and non-practitioners with knowledge can use this measure. Access: Manual, training courses or scale could not be located online to use this measure or reported in paper (Hans, Bernstein & Percansky, 1991) (86).
22	*Parent*–*Infant Pediatric Examination/Pediatric Infant Parent Exam (PIPE)*									
	Fiese et al. (2001) (87) USA	The quality of the parent–infant relationship. With a focus on the reciprocal nature of interactions and how the mother and the infant relate to one another (e.g., positive, or negative affect).	4	Parent(s)-infant ratings (4 items in total): 1. Starting the game: Scale from easy engagement to inappropriate and bizarre engagement (1) 2. Keeping the game going: Easy playfulness to inappropriate play (1) 3. Stopping the game: Gradual cool down to unable to stop game (1) 4. Overall impression of interaction (1) (adaptive: 1, maladaptive: 7)	6-point rating scale (ranging from 1 to 6, ranging from 1 to 7 for the final overall impression subscale). Total score ranges from 4 to 25. Higher scores indicate an increase of problems with interaction e.g., a parent is disengaged or intrusive or infant responds with negative affect. Lower scores reflect more favourable interaction patterns e.g., easy engagement between the parent and infant. At the start, middle and end of the game, the parent–infant interaction is observed and then scored for the degree of interactional reciprocity and positive affect demonstrated.	A brief interactional game without toys (e.g., peekapoo) in a variety of settings. It is designed to be a screening instrument for identification of early signs of difficulties within the parent–infant relationship, for use in primary care settings. Live observations only, videotaped recordings not required. Measure was designed with the intention of being quick to administer, easy to use in a variety of settings and do not require cumbersome testing materials.	6-9 months	Dyad only	The measure was developed informed by transactional and ecological theories (Sameroff, 1993) (88). With an emphasis on the risk of future emotional, cognitive and behavioural difficulties for the infant if there are difficulties in the parent–infant relationship (Bakeman & Brown, 1980) (89). Improving the parent–infant relationship and thereby fostering cognitive development in the infant, requires intervening as early as possible (Achenbach, 1990) (90). The study sample consisted of 117 mothers and their infants between 5.5-9 months old, (infants were either preterm or born full term) during an infant’s routine pediatric exam. Ethnicity described as European American, African American, Asian American or Hispanic American. A range of socioeconomic backgrounds reported. Non-clinical sample.	Costs: Scale free to access in Appendix of Fiese et al. (2001) (87) Training: Training courses unknown. The authors detail reliability assessments as observers are required to obtain acceptable levels of reliability of at least 80% agreement when scoring an observation and that observers were trained in the original development study to rate interactions using the PIPE by the measure developers (Fiese et al., 2001) (87). Access: Manual, or relevant training courses could not be located online to use this measure or reported in Fiese et al. (2001) (87); however, scale is free to access in this paper.
23	*Parent*–*Infant Relational Assessment Tool (PIRAT)*									
	Broughton (2014) (91) UK	A clinical assessment tool for the identification of risk and resilience in the early parent–infant relationship	23	Infant–Parent Scale (12 items in total): 1. Infant’s seeking of contact (1) 2. Responsiveness to contact (1) 3. Responsiveness to stranger (1) 4. Ability to communicate needs (1) 5. Ability to be comforted (1) 6. Quality of contact (7) Parent–Infant Scale (11 items in total): 7. Parent’s initiation of physical contact (1) 8. Parent’s initiation of emotional contact (1) 9. Parent’s playfulness in relation to infant (1) 10. Pleasure in parenting (1) 11. Hostility and blame (1) 12. Quality of contact (6)	3-point rating scale (ranging from 0 to 2). Each subscale is coded as 0 (no concern), 1 (some concern) and 2 (significant concern). Total score ranges from 0 to 46. Consists of two scales, the infant–parent interaction (I-P) and the parent–infant interaction (P-I) scale.	Ease of use for clinicians, reliability and flexibility was prioritised in the design of this measure. Clinicians are instructed to note down when a behaviour is observed on the scale, e.g., whether the behaviour is concerning, somewhat concerning or of no concern. Live observations only, videotaped recordings not required. Clinic or home environment.	0-2 years	Parent and infant only	Measure development informed by literature and research on the impact of difficulties within the parent–infant relationship on infant mental health (Perry et al., 1995) (7). This measure was developed in the Parent–Infant Project (PIP), which was developed at the Anna Freud Centre in 1997. The measure was developed by six psychoanalytic child or group psychotherapists working in PIP, Anna Freud Centre. (Broughton, 2014) (91) The measure was developed to enable clinicians to assess the parent–infant relationship in a clinic or home environment and to identify areas of concern at the earliest opportunity (Broughton, 2014) (91). The study sample consisted of 32 mothers and infants, (aged 0–36 months). No information on ethnicity or socioeconomic status given. Non-clinical sample.	Costs: Unknown. Training: Zoom training offered via Anna Freud Centre – costs and time requirements unknown. Access: Access to scale and manual unknown. Training can be accessed through the following website: https://www.annafreud.org/training/education/under-fives-training-and-events/parent-infant-relational-assessment-tool-pirat-global-scales-training/
24	*Parent*–*Infant Relationship Global Assessment Scale (PIRGAS)*									
	Zero to Three (1994) (92) (Information taken from Aoki et al., 2002; Müller et al., 2013) (93, 94) USA	A focus on assessing the adaptive qualities of the parent–infant relationship.	3	Parent(s)-infant ratings (3 items in total): Behavioural quality of the interaction (1) Affective tone (1) Psychological involvement (1)	Continuous scale ranging from 10 (grossly impaired, disordered relationship) to 50 (distressed relationship) to 90 (well-adapted relationship). Higher scores indicate higher relationship quality. Maximum total score 90, minimum total score of 10.	Observations of mothers and infants playing with toys for 10 min. Clinicians observes the play activity from within the room. Three components of the parent–infant relationship are assessed: behavioural quality of the interaction, affective tone and psycho- logical involvement. Live observations only, videotaped recordings not required. Clinic and laboratory environments.	0-3 years	Dyad only	Measure was developed based on previous literature and research emphasising the consequences of a disrupted parent–infant relationship. Within the Diagnostic Classification of Mental Health and Developmental Disorders of Infancy and Early Childhood (DC: 0-3), to assess qualities of the parent–infant relationship, practitioners are instructed to use the PIRGAS (Aoki et al., 2002) (93). Aoki et al. ‘s (2002) (93) study sample consisted of 53 mothers and their infants (aged 0–2 years). Ethnicities were reported as African-American, White Latina, American Indian and mixed/other. Müller et al.’s (2013) (94) study sample consisted of 84 mother–child dyads. Mean age of the children was 3.88 years and mean age of the mothers was 46. No ethnicity or socioeconomic status discharged. All infants had been admitted to a Child Psychiatric Hospital in Germany. Clinical and non-clinical samples.	Costs: Manual, scale and training requires costs, however costs unknown. Training: requires 4h, costs required but unclear specific amount. Instructions for how to conduct a PIRGAS rating given in the DC:0-3/DC:0-3R manual. Access: DC:0-3R manual costs to access. https://urldefense.com/v3/:https://zerotothree.my.site.com/s/store*/store/browse/cat/a32f40000003gsBAAQ/tiles:;Iw!!PDiH4ENfjr2_Jw!HubGzhnhamOGT9GWXQZBTP4avsuvLGenTYWPZTP0rzLqv-lUI9t9i6RC7kM3ogiuH16Fd2jWlIUluOI9-KYfGlpiFL1f3x6BhfMPX57S$
25	*Tuned-In Parent Rating Scales (TIP-RS)*									
	Priddis and Kane (2013) (95) Australia	A focus on identifying difficulties within the parent–infant relationship, in terms of the parent being ‘tuned in’ to the infant and the quality of the relationship	5	Five aspects of the caregiver and child (5 items in total): 1. Facial expressions in the relationship (1) 2. Use of voice in the relationship (1) 3. Body positioning in the relationship (1) 4. Following the child’s lead (1) 5. Support for exploration and organisation of feelings (1)	5-point rating scale (ranging from 1 to 5). 1, not tuned in, 2, rarely tuned in, 3, sometimes tuned in, to 4 mostly tuned in and 5, very tuned in. The scale requires the coder to attend to five aspects of the behaviour of the caregiver and the infant.	Observational ratings of parent–infant interactions in 15 min of free play and two brief instances of separation and reunion. It is based upon the Ainsworth Strange Situation procedure (Ainsworth et al., 1978) but adapted for use in homes and community clinics. Live observations only, videotaped recordings not required.	0–23 months (< 2 years)	Dyad	Measure development is described to be informed by attachment theory and by literature on maternal sensitivity, in particular the sensitivity scales (Ainsworth et al., 1978; Priddis & Kane, 2013) (95, 96). 88 mothers (aged 22 to 43 years) and their infants (aged 7–23 months). All participants were referred from a community-based early parenting unit in Western Australia. Ethnicity and socioeconomic status not reported. Non-clinical sample.	Costs: Unknown. Training: Unknown. Reliability assessments not reported in Priddis and Kane (2013) (95). Access: Manual, scale or relevant training courses could not be located online to use this measure or reported in Priddis and Kane (2013) (95).

### Overview of identified clinician-rated measures

All measures covered infancy (i.e., from birth to 2 years of age), but some were designed for use with children up to 14 years old, such as the *EAS* (37). The measure with the most severe age restriction was the Family Alliance Assessment Scales for Diaper Change Play (FAAS-DCP) (63), which was suitable for use with infants only within the first three weeks of life. The Parent Infant Interaction Observation Scale (PIIOS) (50) could only be used for a 5-month period from two to seven months.

Only the *AMIS*, *FAAS-DCP*, and *LPICS* were applicable for use with infants under 3 months of age. For use with infants younger than 12 months, only six measures (*BMIS*, *DMC*, *Monadic Phases*, *MRS*, *PIIOS*, and *PIPE*) were applicable. A further seven measures (*Attachment Q sort*, *CIB*, *MACI*, *MRO*, *PIIOS*, *PIOG*, and *PIPE*) were not applicable for use with newborns.

The *AMIS*, *CIB*, *DMC*, *IPSIC*, *MACI*, *PCERA*, and *PIIS* assessed the parent, infant, and the dyad. Ten measures (*Attachment Q sort*, *BMIS*, *CARE-Index*, *EA*S*, M-C ADS*, *Monadic Phases*, *MRS*, *NCATS*, *PIOG*, and *PIRAT*) required clinicians to assess the parent and infant separately. Eight measures (*FAAS-DCP*, *LPICS*, *M-I/TFS*, *MRO*, *PIIOS*, *PIPE*, *PIRGAS*, *and TIP-RS*) assessed only the dyad/triad. All measures included multiple subscales, which ranged from three (*PIRGAS*) to 25 subscales (*AMIS*). The number of items used in the measures ranged from four items (*PIPE*) to 111 items (*MRS*). The length of time required to complete each measure ranged from a “brief” 2-min game in the *PIPE* to 6–8h of observations to complete the *Attachment Q sort.*

Sixteen measures (64%) required the clinician to use videotaped recordings to code the observed relationship, so video recording equipment was required. Seven measures (*Attachment Q sort*, *BMIS*, *PIOG*, *PIPE*, *PIRAT*, *PIRGAS*, and *TIP-*RS) were designed to be completed as live observations of the interactions (no videorecording required) and two measures (*EAS* and *NCATS*) could be completed live or by using videotaped recordings. Thirteen measures (52%) were designed to be completed either in home or clinical (including laboratory) environments. Ten measures were designed to be completed in clinical environments only. Two measures (*CIB* and *IPSIC*) were designed to be completed at the home of the family being assessed.

All 25 measures assessed the parent–infant relationship in terms of expected relationship characteristics, namely, perceived sensitivity and reciprocity (*PIIS)*, sensitivity and responsiveness (*LPICS*, *PIIOS*), reciprocity (*NCATS*, *PIPE*), synchrony (*DMC*), sensitivity and synchrony (*EAS*), mutual responsivity (*MRO*), facial expressions (*MonadicPhases*, *MRS*), quality of the interactions (the *BMIS*, *CIB*, *FAAS-DCP*, *IPSIC*, *MACI*, *PCERA*, *PIOG*, *PIRGAS*, *TIP-RS)*, risk *(PIRAT)* and attachment/attachment behaviours (*Attachment Q sort, CARE-Index, M-C ADS)*. The *AMIS* and the *M-I/TFS* were designed to assess the parent–infant relationship, with a focus on interactions in a feeding context.

In terms of costs, training requirements and access to the measures’ scale, manual and training courses, 15 measures (60%) required the user to be trained in using the measure. However, seven measures (*Attachment Q sort*, *DMC*, *FAAS-DCP*, *LPICS*, *MACI*, *NCATS*, and *PIPE*) required the user to complete training but did not offer further information on how to access this training. The *M-C ADS* required self-study of the published, free to access, manual as training and the *IPSIC* detailed requesting training information from the measure authors. The *AMIS* and *BMIS* did not require the user to be trained to use the measure. For eight measures (*Monadic phases*, *M-I/TFS*, *MRO*, *MRS*, *PCERA*, *PIIS*, *PIOG*, *TIP-RS*), it was unclear if training was required. The *CARE-Index*, *CIB*, *EAS*, *PIIOS*, *PIRAT*, *PIRGAS*, had available training courses accessible via online websites. Of these websites, only three of these measures had costs for these training courses listed, the *CARE-Index* (£850–£1,050), *CIB* ($2,500), and *PIIOS* (£450). For the remaining measures, we could not find information detailing the costs required to access the training courses. When we did find training requirements for measures, the time required to complete the training course ranged from 4h for the *PIRGAS* to nine days for the *CARE-Index*.

Eight measures (32%) and/or their scoring sheets were free to access and accessible in the original development or validation study (*AMIS*, *Attachment Q Sort*, *BMIS*, *IPSIC*, *M-C ADS*, *Monadic Phases*, *MRS*, and *PIPE).* For the *CARE-Index*, *CIB*, *EAS*, *NCATS*, *PIIOS*, *PIRAT*, and *PIRGAS*, costs were involved for manual and scale access, the amount required to access these was unknown for the *EAS*, *NCATS*, *PIRAT*, and *PIRGAS*. The *IPSIC* scale and manual could be requested through the measure authors. The *M-C ADS* had a freely accessible published manual online. The *FAAS-DCP* and *MACI* both have manuals, but access to these could not be located. For fourteen measures, no mention of a manual was made in the studies or could be found online (*AMIS*, *Attachment Q Sort*, *BMIS*, *DMC*, *LPICS*, *Monadic phases*, *M-I/TFS*, *MRO*, *MRS*, *PCERA*, *PIIS*, *PIOG*, *PIPE*, and *TIP-RS*).

### Overview of the quality of measurement properties assessed

Thirty-one studies pertaining to the 25 measures were assessed. **Table 5** provides the overall evidence ratings for each measure, for part A, B, and C. The overall risk of bias of each study was evaluated through the ‘worst score counts’ method. Only one study (76) received an “adequate” rating (evaluating the *M-I/TFS*) for overall risk of bias. Nine studies were rated as “doubtful” (evaluating the *BMIS*, *CARE-Index*, *CIB*, *DMC*, *EAS*, *M-C ADS*, *PIOG*, and *TIP-RS*). Twenty-one of 31 studies (67.7%) received overall scores of “inadequate” in terms of risk of bias. With regards to the quality of evidence reported, only one measure, the *M-I/TFS*, received a “high” rating for quality of evidence reported. Ten measures were assigned “moderate” ratings for at least one measurement property assessed (*BMIS*, *CARE-Index*, *DMC*, *MACI*, *M-I/TFS*, *MRO*, *NCATS*, *PCERA*, *PIIOS*, *TIP-RS*). In terms of final overall evidence, 15 measures were assigned “very low” ratings. Of these, four measures were assigned “very low” ratings for nine out of ten measurement properties (*IPSIC*, *LPICS*, *MRS*, and *PIRAT*). Nine measures were assigned “low” ratings for final overall evidence (*CARE-Index*, *CIB*, *DMC*, *EAS*, *M-C ADS*, *MRO*, *PIIOS*, *PIOG*, *TIP-RS*).

**Table 5:** Overall evidence synthesis for each measure.

	Measure (Study reference)	Category	Total number of studies	Part A (Methodological quality and overall risk of bias)		Part B (Quality appraisal of psychometric properties)	Part C (Overall strength of evidence with GRADE)	Combined/consolidated strength of evidence
1	AMIS (Price, 1983) (58)	Content validity: Relevance	1	D		?	VERY LOW	VERY LOW
		Content validity: Comprehensiveness	1	D		+	VERY LOW	
		Content validity: Comprehensibility	1	D		+	VERY LOW	
		Structural validity	1	D		?	VERY LOW	
		Internal consistency	1	V		?	LOW	
		Reliability	1	D		+	LOW	
		Measurement error	1	D		?	VERY LOW	
		Criterion validity	1	D		?	VERY LOW	
		Hypothesis testing for construct validity	1	I*		?	VERY LOW	
		Responsiveness	1	D		?	VERY LOW	
2	Attachment Q sort (Waters & Dean, 1985) (59)	Content validity: Relevance	1	V		?	VERY LOW	VERY LOW
		Content validity: Comprehensiveness	1	A		+	VERY LOW	
		Content validity: Comprehensibility	1	A		+	VERY LOW	
		Structural validity	1	D		?	VERY LOW	
		Internal consistency	1	A		?	LOW	
		Reliability	1	I		+	VERY LOW	
		Measurement error	1	D		?	VERY LOW	
		Criterion validity	1	D		?	VERY LOW	
		Hypothesis testing for construct validity	1	A		?	LOW	
		Responsiveness	1	I*		?	VERY LOW	
3	BMIS (Kumar & Hipwell, 1996; Stocky, Tonge & Nunn, 1996) (26, 97)	Content validity: Relevance	2	A	A	±	VERY LOW	VERY LOW
		Content validity: Comprehensiveness	2	A	A	-	VERY LOW	
		Content validity: Comprehensibility	2	D	D	+	VERY LOW	
		Structural validity	2	D	D	?	VERY LOW	
		Internal consistency	2	V	V	?	MODERATE	
		Reliability	2	A	D	-	VERY LOW	
		Measurement error	2	D*	D	?	VERY LOW	
		Criterion validity	2	V	D	-	VERY LOW	
		Hypothesis testing for construct validity	2	V	D	-	VERY LOW	
		Responsiveness	2	A	I*	-	VERY LOW	
4	CARE-Index (Crittenden, 1988) (27)	Content validity: Relevance	1	A		+	LOW	LOW
		Content validity: Comprehensiveness	1	D		-	LOW	
		Content validity: Comprehensibility	1	D		+	LOW	
		Structural validity	1	D		?	VERY LOW	
		Internal consistency	1	A		?	LOW	
		Reliability	1	D		?	VERY LOW	
		Measurement error	1	D		?	VERY LOW	
		Criterion validity	1	D*		-	VERY LOW	
		Hypothesis testing for construct validity	1	A		+	MODERATE	
		Responsiveness	1	A		+	LOW	
5	CIB (Stuart et al., 2023) (57)	Content validity: Relevance	1	A		?	LOW	LOW
		Content validity: Comprehensiveness	1	A		?	LOW	
		Content validity: Comprehensibility	1	D		?	LOW	
		Structural validity	1	A		-	LOW	
		Internal consistency	1	V		+	MODERATE	
		Reliability	1	A		+	LOW	
		Measurement error	1	D		?	VERY LOW	
		Criterion validity	1	A		?	LOW	
		Hypothesis testing for construct validity	1	A		-	LOW	
		Responsiveness	1	D*		-	VERY LOW	
6	DMC (Censullo et al., 1987) (61)	Content validity: Relevance	1	A		+	LOW	LOW
		Content validity: Comprehensiveness	1	D		+	LOW	
		Content validity: Comprehensibility	1	D		+	LOW	
		Structural validity	1	D		?	VERY LOW	
		Internal consistency	1	D		?	VERY LOW	
		Reliability	1	D		-	VERY LOW	
		Measurement error	1	D*		?	VERY LOW	
		Criterion validity	1	A		-	LOW	
		Hypothesis testing for construct validity	1	A		+	MODERATE	
		Responsiveness	1	A		?	LOW	
7	EAS (Aran et al., 2022) (62)	Content validity: Relevance	1	D		?	LOW	LOW
		Content validity: Comprehensiveness	1	D		?	LOW	
		Content validity: Comprehensibility	1	D		+	LOW	
		Structural validity	1	V		-	VERY LOW	
		Internal consistency	1	D		?	LOW	
		Reliability	1	D		+	LOW	
		Measurement error	1	D		?	VERY LOW	
		Criterion validity	1	D		?	VERY LOW	
		Hypothesis testing for construct validity	1	V		-	LOW	
		Responsiveness	1	D*		-	VERY LOW	
8	FAAS-DCP (Rime et al., 2018) (63)	Content validity: Relevance	1	D		?	VERY LOW	VERY LOW
		Content validity: Comprehensiveness	1	D		+	VERY LOW	
		Content validity: Comprehensibility	1	D		?	VERY LOW	
		Structural validity	1	A		+	LOW	
		Internal consistency	1	V		+	LOW	
		Reliability	1	A		-	VERY LOW	
		Measurement error	1	D		?	VERY LOW	
		Criterion validity	1	A		-	VERY LOW	
		Hypothesis testing for construct validity	1	A		-	VERY LOW	
		Responsiveness	1	I*		-	VERY LOW	
9	IPSIC (Baird et al., 1992) (66)	Content validity: Relevance	1	A		?	VERY LOW	VERY LOW
		Content validity: Comprehensiveness	1	A		+	VERY LOW	
		Content validity: Comprehensibility	1	A		+	VERY LOW	
		Structural validity	1	D		?	VERY LOW	
		Internal consistency	1	D		?	VERY LOW	
		Reliability	1	D		-	VERY LOW	
		Measurement error	1	D		?	VERY LOW	
		Criterion validity	1	I		?	VERY LOW	
		Hypothesis testing for construct validity	1	A		-	LOW	
		Responsiveness	1	I*		?	VERY LOW	
10	LPICS (Beatty et al., 2011) (67)	Content validity: Relevance	1	A		+	VERY LOW	VERY LOW
		Content validity: Comprehensiveness	1	I		?	VERY LOW	
		Content validity: Comprehensibility	1	D		?	VERY LOW	
		Structural validity	1	D		?	VERY LOW	
		Internal consistency	1	D		?	VERY LOW	
		Reliability	1	D		-	VERY LOW	
		Measurement error	1	D		?	VERY LOW	
		Criterion validity	1	D		?	LOW	
		Hypothesis testing for construct validity	1	D		-	VERY LOW	
		Responsiveness	1	I*		-	VERY LOW	
11	MACI (Wan et al., 2017) (69)	Content validity: Relevance	1	D		+	VERY LOW	VERY LOW
		Content validity: Comprehensiveness	1	D		?	VERY LOW	
		Content validity: Comprehensibility	1	D		?	VERY LOW	
		Structural validity	1	D		?	VERY LOW	
		Internal consistency	1	A		?	LOW	
		Reliability	1	I		-	VERY LOW	
		Measurement error	1	I*		?	VERY LOW	
		Criterion validity	1	D		-	VERY LOW	
		Hypothesis testing for construct validity	1	A		-	LOW	
		Responsiveness	1	V		+	MODERATE	
12	M-C ADS (Cárcamo et al., 2014; Nóblega et al., 2019) (73, 74)	Content validity: Relevance	2	A	A	+	LOW	LOW
		Content validity: Comprehensiveness	2	A	A	?	LOW	
		Content validity: Comprehensibility	2	D	A	?	LOW	
		Structural validity	2	D	D	?	VERY LOW	
		Internal consistency	2	D	D	?	VERY LOW	
		Reliability	2	D	D	-	VERY LOW	
		Measurement error	2	D	A	-	LOW	
		Criterion validity	2	A	A	-	LOW	
		Hypothesis testing for construct validity	2	V	A	-	LOW	
		Responsiveness	2	D*	D*	-	VERY LOW	
13	M-I/TFS (Chatoor et al., 1997) (76)	Content validity: Relevance	1	A		+	MODERATE	MODERATE
		Content validity: Comprehensiveness	1	V		+	MODERATE	
		Content validity: Comprehensibility	1	A		+	MODERATE	
		Structural validity	1	A		?	LOW	
		Internal consistency	1	A		?	LOW	
		Reliability	1	A		+	MODERATE	
		Measurement error	1	A		?	LOW	
		Criterion validity	1	A*		?	LOW	
		Hypothesis testing for construct validity	1	V		+	HIGH	
		Responsiveness	1	V		+	HIGH	
14	Monadic Phases (Matias, Cohn & Ross, 1989; Tronick, Als & Brazleton, 1980) (56, 98)	Content validity: Relevance	2	D	D	+	VERY LOW	VERY LOW
		Content validity: Comprehensiveness	2	D	D	-	VERY LOW	
		Content validity: Comprehensibility	2	D	D	?	VERY LOW	
		Structural validity	2	D	D	?	VERY LOW	
		Internal consistency	2	D	D	?	VERY LOW	
		Reliability	2	D	D	?	LOW	
		Measurement error	2	D	D	?	VERY LOW	
		Criterion validity	2	V	D	-	LOW	
		Hypothesis testing for construct validity	2	A	A	-	VERY LOW	
		Responsiveness	2	I*	I*	-	VERY LOW	
15	MRS (Tronick et al., 1978) (77)	Content validity: Relevance	1	D		?	VERY LOW	VERY LOW
		Content validity: Comprehensiveness	1	D		?	VERY LOW	
		Content validity: Comprehensibility	1	D		?	VERY LOW	
		Structural validity	1	D		?	VERY LOW	
		Internal consistency	1	D		?	VERY LOW	
		Reliability	1	D		?	VERY LOW	
		Measurement error	1	I		?	VERY LOW	
		Criterion validity	1	I*		?	VERY LOW	
		Hypothesis testing for construct validity	1	D		?	VERY LOW	
		Responsiveness	1	A		?	LOW	
16	MRO (Aksan, Kochanska &Ortmann, 2006) (78)	Content validity: Relevance	1	D		+	VERY LOW	LOW
		Content validity: Comprehensiveness	1	D		?	VERY LOW	
		Content validity: Comprehensibility	1	D		?	VERY LOW	
		Structural validity	1	V		-	LOW	
		Internal consistency	1	V		?	MODERATE	
		Reliability	1	D		+	LOW	
		Measurement error	1	D		?	VERY LOW	
		Criterion validity	1	I*		?	VERY LOW	
		Hypothesis testing for construct validity	1	A		-	LOW	
		Responsiveness	1	A		-	LOW	
17	NCATS (Byrne & Keefe, 2003; Gross et al., 1993) (81, 82)	Content validity: Relevance	2	D	D	±	VERY LOW	VERY LOW
		Content validity: Comprehensiveness	2	A	D	?	VERY LOW	
		Content validity: Comprehensibility	2	A	D	±	VERY LOW	
		Structural validity	2	D	D	?	VERY LOW	
		Internal consistency	2	V	V	?	MODERATE	
		Reliability	2	D	D	-	VERY LOW	
		Measurement error	2	D	D	-	LOW	
		Criterion validity	2	V	I	-	VERY LOW	
		Hypothesis testing for construct validity	2	I*	D	-	VERY LOW	
		Responsiveness	2	A	I*	-	VERY LOW	
18	PCERA (Clark, 1999) (83)	Content validity: Relevance	1	D		?	VERY LOW	VERY LOW
		Content validity: Comprehensiveness	1	D		?	VERY LOW	
		Content validity: Comprehensibility	1	D		?	VERY LOW	
		Structural validity	1	A		+	MODERATE	
		Internal consistency	1	V		+	MODERATE	
		Reliability	1	D		-	LOW	
		Measurement error	1	D		?	VERY LOW	
		Criterion validity	1	D		?	VERY LOW	
		Hypothesis testing for construct validity	1	A		?	LOW	
		Responsiveness	1	I*		?	VERY LOW	
19	PIIOS (Svanberg, Barlow & Tigbe, 2013) (50)	Content validity: Relevance	1	A		?	VERY LOW	LOW
		Content validity: Comprehensiveness	1	A		?	VERY LOW	
		Content validity: Comprehensibility	1	A		?	VERY LOW	
		Structural validity	1	D		?	VERY LOW	
		Internal consistency	1	V		?	MODERATE	
		Reliability	1	D		-	VERY LOW	
		Measurement error	1	D		?	VERY LOW	
		Criterion validity	1	D		+	MODERATE	
		Hypothesis testing for construct validity	1	I		-	VERY LOW	
		Responsiveness	1	I*		-	VERY LOW	
20	PIIS (Clark & Seifer, 1985) (28)	Content validity: Relevance	1	D		+	VERY LOW	VERY LOW
		Content validity: Comprehensiveness	1	D		?	VERY LOW	
		Content validity: Comprehensibility	1	D		?	VERY LOW	
		Structural validity	1	D		?	VERY LOW	
		Internal consistency	1	A		?	LOW	
		Reliability	1	D		?	VERY LOW	
		Measurement error	1	D		?	VERY LOW	
		Criterion validity	1	I*		?	VERY LOW	
		Hypothesis testing for construct validity	1	D		?	VERY LOW	
		Responsiveness	1	V		?	LOW	
21	PIOG (Bernstein et al., 2005; Hans, Bernstein & Percansky, 1991) (86, 99)	Content validity: Relevance	2	A	A	?	LOW	LOW
		Content validity: Comprehensiveness	2	V	D	+	LOW	
		Content validity: Comprehensibility	2	V	A	+	LOW	
		Structural validity	2	A	D	+	LOW	
		Internal consistency	2	V	V	-	LOW	
		Reliability	2	D	D	-	VERY LOW	
		Measurement error	2	D	I	-	VERY LOW	
		Criterion validity	2	A	D	-	VERY LOW	
		Hypothesis testing for construct validity	2	A	I	?	VERY LOW	
		Responsiveness	2	D*	I*	?	VERY LOW	
22	PIPE (Fiese et al., 2001) (87)	Content validity: Relevance	1	D		?	VERY LOW	VERY LOW
		Content validity: Comprehensiveness	1	D		-	VERY LOW	
		Content validity: Comprehensibility	1	D		?	VERY LOW	
		Structural validity	1	D		?	VERY LOW	
		Internal consistency	1	D		?	VERY LOW	
		Reliability	1	D		+	LOW	
		Measurement error	1	D		?	VERY LOW	
		Criterion validity	1	I*		?	VERY LOW	
		Hypothesis testing for construct validity	1	A		-	LOW	
		Responsiveness	1	D		-	VERY LOW	
23	PIRAT (Broughton, 2014) (91)	Content validity: Relevance	1	D		?	VERY LOW	VERY LOW
		Content validity: Comprehensiveness	1	V		+	VERY LOW	
		Content validity: Comprehensibility	1	V		+	VERY LOW	
		Structural validity	1	D		?	VERY LOW	
		Internal consistency	1	V		?	LOW	
		Reliability	1	I		-	VERY LOW	
		Measurement error	1	I		?	VERY LOW	
		Criterion validity	1	D		-	VERY LOW	
		Hypothesis testing for construct validity	1	I		?	VERY LOW	
		Responsiveness	1	I*		?	VERY LOW	
24	PIRGAS (Aoki et al., 2002; Müller et al., 2013) (93, 94)	Content validity: Relevance	2	D	D	?	VERY LOW	VERY LOW
		Content validity: Comprehensiveness	2	D	A	?	VERY LOW	
		Content validity: Comprehensibility	2	D	D	?	VERY LOW	
		Structural validity	2	D	D	?	VERY LOW	
		Internal consistency	2	D	D	?	VERY LOW	
		Reliability	2	D	D	?	LOW	
		Measurement error	2	I*	I*	?	VERY LOW	
		Criterion validity	2	A	A	-	LOW	
		Hypothesis testing for construct validity	2	A	A	-	LOW	
		Responsiveness	2	D	D	-	VERY LOW	
25	TIP-RS (Priddis & Kane, 2013) (95)	Content validity: Relevance	1	A		?	LOW	LOW
		Content validity: Comprehensiveness	1	A		+	LOW	
		Content validity: Comprehensibility	1	A		+	LOW	
		Structural validity	1	A		-	LOW	
		Internal consistency	1	V		?	MODERATE	
		Reliability	1	D		-	LOW	
		Measurement error	1	D*		?	VERY LOW	
		Criterion validity	1	V		-	LOW	
		Hypothesis testing for construct validity	1	A		?	LOW	
		Responsiveness	1	A		?	LOW	

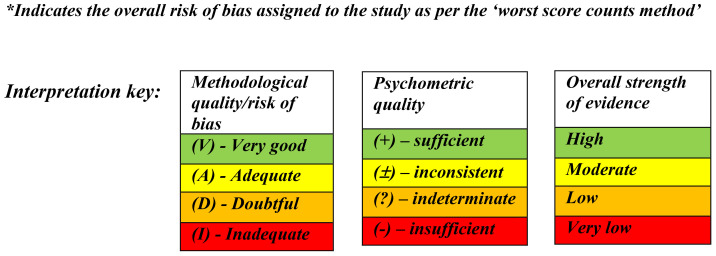

### Assessment of validity

#### Content validity

Due to many of the studies having very different scores for content validity or no content validity studies identified, it was important to report the relevance, comprehensiveness and comprehensibility ratings separately (see Terwee et al.’s (42) criteria for assessing content validity). Fifteen of 31 studies (48.4%) reported evaluating the “relevance” of the measure’s items. Only one study received a “very good” rating for “relevance” among participants using the *Attachment Q sort* (62) in terms of methodological quality. Fourteen studies were rated “adequate” for methodological quality and the remaining 16 studies were rated “doubtful” due to not enough evidence being given as to whether “relevance” was assessed by the study authors.

With regard to “comprehensiveness” 14 studies (45.2.%) reported evaluating this aspect: three studies received a “very good” rating (*M-I/TFS* (77), *PIOG* (99), and *PIRAT* (91)) in terms of methodological quality. Eleven studies were rated “adequate,” 16 studies received a “doubtful” rating and one study (*LPICS* (68)) received an “inadequate” rating. Ten studies (32.3%) reported assessing “comprehensibility,” but only two studies were rated “very good” (*PIOG* (99), *PIRAT* (91)) for methodological quality. Eight studies were rated “adequate” and the remaining 21 studies were assigned “doubtful” ratings due to not enough information being given by the study authors to assign any higher rating.

In terms of quality appraisal of the psychometric properties in the reported results, relevance, comprehensiveness and comprehensibility were again evaluated separately. With regard to the quality appraisal of findings for relevance, nine measures received “sufficient” (+) ratings (*CARE-Index*, *DMC*, *LPICS*, *MACI*, *M-C ADS*, *M-I/TFS*, *Monadic Phases*, *MRO*, *PIIS*). Two measures received “inconsistent” ratings (±), the *BMIS* and *NCATS.*

The “inconsistent” ratings arose because relevance, comprehensiveness, and/or comprehensibility was “sufficient” for one study but “insufficient” for another, so the ratings of content validity differed between two studies evaluating the same measure. The remaining 14 measures received “indeterminate” ()? ratings, due to many of the studies failing to report enough information in the results to meet a “sufficient” rating. In terms of the quality appraisal of results for “comprehensiveness,” nine measures received “sufficient” ratings (*AMIS, Attachment Q sort, DMC, FAAS-DCP, IPSIC, M-I/TFS, PIOG, PIRAT* and *TIP-RS*). Four measures received “insufficient” ratings (*BMIS*, *CARE-Index*, *Monadic Phases*, and *PIPE*), whereas the remaining 12 measures received “indeterminate” ratings. Finally, in terms of “comprehensibility,” 11 measures (*AMIS, Attachment Q sort*, *BMIS*, *CARE-Index*, *DMC*, *EAS*, *IPSIC*, *M-I/TFS*, *PIOG*, *PIRAT*, and *TIP-RS*) received “sufficient” ratings. One measure (*NCATS*) received an “inconsistent” rating. The remaining 13 measures received “indeterminate” ratings.

In the final step, the scores assigned for both methodological quality and psychometric properties of a measure were rated using the GRADE approach. As per COSMIN criteria, if a study received an “inadequate” risk of bias rating, then the measure evaluated in that study received a “very low” rating in terms of the GRADE for relevance, comprehensiveness and comprehensibility. If the study was rated as of “doubtful” quality, the content validity ratings were of “low” quality. Therefore, only one measure, the *M-I/TFS*, was rated as “moderate” quality of evidence for content validity due to receiving an “adequate” overall score for risk of bias. Seven measures (the *CARE-Index*, *CIB*, *DMC*, *EAS*, *M-C ADS*, *PIOG*, and *TIP-RS*) were rated as “low” quality evidence and 17 measures were rated as “very low” quality of evidence for relevance, comprehensiveness and comprehensibility according to the GRADE approach.

#### Structural validity

Two studies (evaluating the *EAS* and *MRO*) were rated “very good” for methodological quality, and six studies (evaluating the *CIB*, *FAAS-DCP*, *M-I/TFS*, *PCERA*, *PIOG*, *TIP-RS*) were rated as “adequate” due to most of these studies using EFA. Studies using EFA could only be rated as “adequate” rather than “very good” for methodological quality. The remaining studies were rated as “doubtful” due to not providing information pertaining to the assessment of or consideration of structural validity.

Structural validity was assessed in studies for only seven of the 25 measures (28%). Only the *FAAS-DCP, PCERA* and *PIOG* were assigned a “sufficient” rating for quality appraisal; EFA was used to assess their structural validity. The *CIB*, *EAS, MRO* and *TIP-RS* were assigned “insufficient” ratings. All four of these measures had studies reporting on structural validity of the measure using CFA; all four reported results were “insufficient” for the COSMIN criteria. The remaining 18 measures were assigned “indeterminate” ratings due to not reporting enough information on the structural validity of the measure to meet the criterion for either a “sufficient” of “insufficient” rating. The quality of the evidence ranged from “moderate” to “very low” for this measurement property.

#### Hypothesis testing for construct validity

Seventeen measures of the 25 (68%) had studies reporting information regarding construct validity. Four studies (assessing the *BMIS*, *EAS*, *M-C ADS*, *M-I/TFS*) received “very good” ratings for methodological quality and 17 studies received “adequate” ratings. Five studies (assessing the *AMIS*, *NCATS*, *PIIOS*, *PIOG*, *PIRAT)* received “inadequate” ratings. The remaining five studies received “doubtful” ratings.

In terms of quality appraisal of the psychometric properties only the *CARE-Index, DMC* and the *M-I/TFS* were assigned “sufficient” ratings. Fourteen measures were assigned “insufficient” ratings and the remaining eight measures were assigned “indeterminate” ratings (if hypotheses could not be defined by the review team). Gradings of “high” to “very low” were given for the quality of evidence for this measurement property.

#### Criterion validity

The assessment of criterion validity was reported in studies for 13 measures (52%). Four studies were assigned “very good” ratings for methodological quality (the *BMIS*, *Monadic Phases*, *NCATS*, and *TIP-RS*). Nine studies were assigned “adequate” ratings for methodological quality and six studies were assigned “insufficient” ratings. The remaining 12 studies were assigned “doubtful” ratings.

However, with regard to then appraising the measures’ reported psychometric properties, only the *PIIOS* was assigned a “sufficient” rating for criterion validity. Twelve measures were assigned “insufficient” ratings and the remaining 12 measures were assigned an “indeterminate” rating. The quality of the evidence was graded “moderate” to “very low” for this measurement property.

### Assessment of reliability

#### Internal consistency

In terms of internal consistency, 14 studies were assigned “very good” ratings and five studies were assigned “adequate” ratings regarding methodological quality. The remaining 12 studies were assigned “doubtful” ratings, with no studies receiving a rating of “inadequate.”

Internal consistency was reported in studies for 16 of the 25 measures (64%). Despite this, the COSMIN criteria stipulate that outcome measures that do not demonstrate at least “low” evidence of “sufficient” validity can be rated as “indeterminate” only. Therefore, only the *CIB*, *FAAS-DCP* and *PCERA* were rated as “sufficient” for psychometric evidence for internal consistency, the *PIOG* was assigned ratings of “insufficient,” with the remaining 21 measures rated as “indeterminate.” For internal consistency, the quality of the evidence was graded “moderate” to “very low.”

#### Reliability

Reliability of the measures was reported in studies for 20 of the 25 measures (80%). Only four studies received “adequate” ratings for methodological quality (studies reporting on the *BMIS*, *CIB*, *FAAS-DCP*, and *M-I/TFS*). Three studies reporting on the *Attachment Q sort*, *MACI*, and *PIRAT* were assigned “inadequate” ratings. The remaining studies received “doubtful” ratings.

Regarding quality appraisal of psychometric properties, seven measures were assigned “sufficient” ratings, comprising the *AMIS*, *Attachment Q Sort*, *CIB*, *EAS*, *M-I/TFS*, *MRO*, and *PIPE.* Thirteen measures were assigned “insufficient” ratings. The remaining five measures (*CARE-Index*, *Monadic Phases*, *MRS*, *PIIS,* and *PIRGAS*) were assigned “indeterminate” ratings. The quality of the evidence was graded “moderate” to “very low” for this measurement property.

#### Measurement error

Three measures (12%) had studies reporting on measurement error. With regard to methodological quality, only two studies were rated as “adequate” (*M-C ADS* and *M-I/TFS*). Six studies were rated as “inadequate” (for the *MACI*, *MRS*, *PIOG*, *PIRAT*, and *PIRGAS*). The remaining 23 studies were assigned ratings of “doubtful” for methodological quality.

With regard to quality appraisal of psychometric properties ratings, three measures (the *M-C ADS*, *NCATS*, and *PIOG*) were assigned “insufficient” ratings as per the COSMIN criteria for measurement error. The remaining 22 measures were assigned “indeterminate” ratings. The quality of the evidence was graded “moderate” to “very low” for this measurement property.

#### Responsiveness

In terms of responsiveness, three studies reporting on the *MACI, M-I/TFS* and *PIIS* were assigned “very good” ratings for methodological quality. Seven studies received “adequate” ratings (for the *BMIS*, *CARE-Index*, *DMC*, *MRS*, *MRO*, *NCATS*, and the *TIP-RS*) and 12 studies received “inadequate” ratings for methodological quality. The remaining nine studies were rated “doubtful” quality.

Fifteen of the 25 measures (60%) had studies reporting information for responsiveness. Only the *CARE-Index*, *MACI*, and the *M-I/TFS* were assigned “sufficient” ratings for quality appraisal of the reported psychometric properties. The other 12 measures were deemed to have “insufficient” information to meet the COSMIN criteria for a rating of “sufficient.” The remaining ten measures were assigned “indeterminate” ratings. Finally, with regard to responsiveness the quality of the evidence was graded “high” to “very low.”

#### Inter-rater reliability

To ensure inter-rater reliability and quality of the ratings, an independent researcher undertook quality ratings for 25% of identified papers describing the measures. An exact agreement of 87.5% was achieved for the quality ratings, with any discrepancies resolved through discussion.

## Discussion

This review systematically identified and examined 25 clinician-rated parent–infant assessments and comprehensively examine their psychometric properties and their overall quality, informed by the COSMIN criteria. A previous review by Munson and Odom in 1996 (34) identified 17 clinician-rated parent–infant assessments, of which only five met inclusion criteria for this current review. A review completed by Bagner et al. in 2012 (35) identified four clinician-rated parent–infant assessments, of which three were included in this review. In 2015, Lotzin et al. (32) reviewed 24 clinician-rated parent–infant assessment measures, of which eight measures were not included in this systematic review. These differences could be attributed to differences in inclusion and exclusion criteria; for example, Munson and Odom (34) drew on book chapters for information, rather than peer-reviewed journals. Bagner et al. (35) did not offer detailed information on their search strategy or their inclusion/exclusion criteria. Lotzin et al. (32) only included measures with more than one study outlining information that described the development and/or validation of the measure, whereas this current review included measures even if only one relevant study was identified. The differences in methods between these reviews and the current review are important to consider because it may be due to these differences as to why different assessment measures were ultimately included in this review.

The measures identified assessed the parent–infant relationship in very young babies, across the age range from birth to two years of age and for specific contexts, such as during a feed. Two measures (*AMIS*, *LPICS*) could be used in a short time period only, namely, from birth to 3 months old. The *FAAS-DCP* and the *PIIOS* had very strict periods of use from birth to 3 weeks and 2–7 months, respectively. The *AMIS* and *M-I/TFS* could be used in specific feeding contexts only. Thus, these 6 measures can only be used in very specific contexts, so they may not be applicable for wider implementation in perinatal services.

All 25 measures assessed the parent–infant relationship in terms of expected relationship characteristics, with the most common focus of the measure being the perceived quality of the parent–infant interaction (nine measures focused on this). Over half (60%) of the measures required the clinician to complete further training to use the measure; however, training courses or information could not be located for seven of these measures. Furthermore, only eight measures offered free access to the scales and no access to a manual could be found in the included studies or online, for 14 measures.

The COSMIN criteria are considered stringent (39); as a result, some measures, for which adequate psychometric properties were reported, were assigned scores that fell short of the stringent COSMIN requirements. The *M-I/TFS*, followed by the *TIP-RS* and *CIB* demonstrated the most promising evidence overall. However, the measure that demonstrated the best psychometric properties is limited in its uses due to being used in a feeding context only. Thus, its utility across other specific contexts (e.g., structured play, i.e., a play interaction guided or structured by the caregiver) is limited. Twenty-one of 31 studies (67.7%) received overall scores of “inadequate” in terms of risk of bias. Structural validity was reported variably across the studies. All studies reporting use of EFA to assess structural validity were assigned “sufficient” ratings; all studies that used CFA to report on structural validity were assigned “insufficient” ratings. The most frequently assessed measurement property was reliability: 80% of studies reported this. With regard to internal consistency, only three measures (*CIB*, *FAAS-DCP*, *PCERA*) ultimately received ratings of “sufficient” due to the remaining measures not meeting the COSMIN criteria of atleast low structural validity to receive a rating of “sufficient.” No measure received a “sufficient” rating in terms of measurement error and only one measure (*PIIOS*) was assigned a “sufficient” rating for criterion validity. With regard to the strength of evidence, the majority of measures were assigned “very low” and “low” ratings using the GRADE approach. Only one measure scored “moderate” for overall evidence, the *M-I/TFS*. The *M-I/TFS* was also the only measure to be scored “high” in two measurement properties, according to the GRADE approach. However, this measure still scored “low” ratings for strength of evidence across four other measurement properties. Consequently, our recommendations regarding the use of each identified measure are cautiously provided and clinicians should be aware of the quality disparities across assessment measures. These novel findings are important, because they extend knowledge as to the quality of the parent–infant assessments that are in use. This review highlights the importance of transparency in reporting and the need for more detailed accounts of psychometric properties of measures.

### Considerations relating to content validity

Content validity has been argued to be the most important psychometric property (38, 42); the relevance, comprehensiveness, and comprehensibility of a measure can be an important contributing factor when a clinician is deciding whether to use a measure for clinical or research purposes (42). Content validity was most often demonstrated within studies by a variable description of a theory-driven method or a review of relevant literature driving the development of individual items or subscales but detail was often lacking.Authors rarely reported on involving professionals or participants in the target population in the development of the measure. Many authors failed to mention necessary details of how they had conducted any evaluations of content validity, and as a result, applying the stringent COSMIN criteria for content validity resulted in many studies being rated as “low” or “very low” for overall quality of evidence. This is an important finding: experts (by profession, or via lived experience) should be involved in the development or adaptation of measures to improve content validity. More detailed evaluations of the content validity of these assessment measures should be prioritised in future research to increase confidence in the measure (100, 101).

### Considerations relating to structural validity

Although validity evidence based on internal structure is essential to support the use of an outcome measure (102), 23 studies (74.2%) were assigned ratings of “doubtful” for this in terms of methodological quality. Only three measures, the *FAAS-DCP, PCERA* and *PIOG*, showed “sufficient” evidence of structural validity with “moderate” or “low” quality of evidence. All three used EFA to assess structural validity. Of the four measures that showed “insufficient” evidence (the *CIB*, *EAS*, *MRO*, and *TIP-RS*), all used CFA to assess structural validity. This finding is important as it adds more weight to Schmitt et al. (103): misconceptions exist among researchers whether to use CFA, EFA, or a combination of both factor analytic approaches and many researchers often mistakenly use CFA methods when EFA may be more appropriate.

### Considerations relating to construct validity

Only the *CARE-Index, DMC* and *M-I/TFS* were assigned “sufficient” ratings in terms of quality appraisal, despite 17 measures (68%) reporting information regarding this measurement property. Developing more rigorous assessments of construct validity is important because misattribution or misidentification of the cause of or the effects of the measure can lead to inaccuracies in measurement (104). Therefore, we identified a need to comprehensively establish construct validity of outcome measures and improve the transparent reporting of construct validity in order for clinicians and researchers to make accurate and informed decisions.

### Considerations relating to criterion validity

Although many authors assessed criterion validity by comparing the measure against a “gold-standard” clinician-rated measure of the parent–infant relationship, such as the CARE-Index (as described in Svanberg, Barlow and Tigbe (50), only the *PIIOS* was assigned a “sufficient” rating for criterion validity. Many authors demonstrated their measure’s efficacy in discriminating between high- and low-risk participants or reported on the measure’s constructs being correlated with other similar constructs. Furthermore, many authors reported criterion validity when only assessing specific aspects of criterion validity, such as hypothesis testing, convergent or discriminant validity. Authors rarely assessed predictive validity, that is, whether scores predicted future developmental outcomes, a significant omission (32).

### Considerations relating to internal consistency

Authors typically reported adequate levels of internal consistency. Fourteen studies were assigned “very good” ratings, and five studies were assigned “adequate” ratings regarding methodological quality for internal consistency. However, due to “very low” ratings for sufficient structural validity for many studies, only the *CIB, FAAS-DCP* and *PCERA* were rated as “sufficient” for internal consistency. All studies used Cronbach’s alpha. However, when it is used to assess items that cover a broad or more complex topic, it has been suggested that Cronbach’s alpha may underestimate the internal consistency of the measure (105) and some researchers have suggested alphas should not be interpreted as a measure of internal consistency (106).

### Considerations relating to reliability

Twenty of the 25 measures (80%) had studies that reported on the reliability of the measure, but only seven measures were assigned “sufficient” ratings for the quality appraisal of the reported reliability results. While most studies reported on inter-rater reliability, no studies explicitly reported on assessing intra-rater reliability. Intra-rater reliability estimates are also important, because a researcher can assess if there are any practice effects associated with clinicians becoming familiar with the outcome measure (107).

### Considerations relating to measurement error

Only three measures (12%, *M-C ADS*, *NCATS*, and *PIOG*) had studies that reported on measurement error, and they were ultimately assigned “insufficient” ratings. The remaining 22 measures were rated “indeterminate.” On reflection, many of the studies did report adequate percentage agreement (>80%) but failed to explicitly define the minimal important change (MIC), meaning they were assigned ratings of “indeterminate” as per COSMIN criteria. It is important to define the MIC because if the reported measurement error in a study is smaller than the MIC, it may be possible for researchers to identify and distinguish clinically important changes from measurement error with a greater amount of certainty (108). Additionally, many studies failed to report on sensitivity, specificity and/or accuracy, which led to ratings of “doubtful” for 74.2% of studies for methodological quality. Further information is required on the sensitivity, specificity and accuracy in order for clinicians and researchers to be able to use the measures to identify parent–infant relationships at risk of breaking down or having long term consequences for infant mental health (32).

### Considerations relating to responsiveness

Fifteen measures (60%) had studies reporting information on responsiveness of the measure. However, only the *CARE-Index, MACI* and *M-I/TFS* were assigned “sufficient” ratings in terms of responsiveness. The other 12 measures were deemed to be “insufficient.” Few studies reported longitudinal parent–infant relationship data. Responsiveness is important to establish the ability of a measure to detect change over time i (38, 39, 109). Thus, more research is needed to fully establish the responsivity of these measures in order to continue monitoring changes over time within the parent–infant relationship.

### Considerations relating to the use of the COSMIN guidelines

The COSMIN criteria are regarded as the most stringent and comprehensive to apply to studies due to the multi-step process outlined previously, which is considered a strength of this review (39). Despite this, as highlighted by Jewell et al. (110), these stringent cutoff scores can lead to important information being overlooked. In some cases in this review, the reported results were very close to values defined as “sufficient” indicating a positive result, but were rated as “insufficient” due to not meeting the stringent COSMIN cutoff values. Additionally, the COSMIN checklist was used to assess the study’s risk of bias using the “worst score counts” method, meaning that even one flaw in a study would result in a “doubtful” or “inadequate” rating overall, despite demonstrating “very good” evidence in other methodological aspects. Jewell et al. (110) argued that this method results in the overall reported methodological quality of a study as potentially not being an accurate reflection of the study’s risk of bias and perhaps leading to an underestimation of the adequacy of reported measurement properties., Poor risk of bias ratings often stemmed from a lack of information reported by authors, meaning they did not meet the stringent COSMIN criteria. It should be noted, however, that 64.5% of studies were published before the COSMIN criteria were available.

Additionally, 16% of the measures were developed or validated with a sample of 50 or fewer participants leading to possible imprecisions. Inadequate sample sizes, in terms of COSMIN criteria, and inadequate risk of bias scores often affected the overall grading of the quality of evidence. However, the subsequent use of such a measure would not necessarily be based on robust and strong psychometric evidence.

### Strengths and limitations

One major strength of this review is its comprehensiveness. More than 13,000 records across all publication years in five databases were screened. This approach reduced the likelihood of missing any relevant studies and resulted in a robust approach to reviewing all studies that used or reported on assessment measures of the parent–infant relationship. Stage 1 enabled the identification of a high number of parent–infant assessment measures.

Earlier reviews (32, 34, 35) published in, and prior to 2015, included a smaller number of measures and were not assessed as comprehensively since they were not informed by the COSMIN criteria.

Some limitations to this review are acknowledged. Validity and reliability evidence was based only on studies in peer-reviewed journals that psychometrically evaluated or described the development of the measure. Other literature (e.g., book chapters and theses) was excluded; thus, other relevant evidence for the included measures may exist but was not included. Additionally, this review excluded measures that were not suitable for infants aged 0–2 years. Consequently, we may have underreported the breadth of parent–infant assessment measures available for clinicians and researchers to use.

Furthermore, we excluded non-English language studies due to the limited time and resources available. Thus, the presence of possible language and location biases is acknowledged (111). However, only two identified measures (one in each stage) were excluded due to the relevant studies being in a non-English language. Additionally, alternatives to the COSMIN tools when assessing the psychometric properties of outcome measures exist, such as the Evaluating the Measurement of Patient-Reported Outcomes Tool (EMPRO) (112), a 39-item standardised assessment tool that has been used to review psychometric properties of measures in other systematic reviews (113) and the Francis tool (114), which uses an 18-item checklist to appraise the psychometric properties of instruments (115).

We acknowledge a further limitation of this review in terms of the potential exclusion of literature that might have examined construct validity: the authors of those studies might not have specifically stated that they intended to fully validate the measure (e.g., they might have used a method to investigate the relationship between two measures rather than examined the validity of a particular measure).Therefore, it is possible that that this aspect was not reported because the authors did not have that as their primary aim.

### Implications for future research and practical recommendations

Of the final 25 parent–infant assessment measures that were first identified and then evaluated in this review, the majority (76%) had only one suitable study describing or evaluating the measure’s development and/or psychometric properties. Hence, further research demonstrating each measure’s reliability and validity would be useful for clinicians.

As suggested in a review by Lotzin et al. (32), parent–infant assessment measures could further refine the constructs, subscales or items used. Five measures identified in this review (*Attachment Q sort*, *Monadic Phases*, *MRS*, *NCATS*, and *PCERA*) included more than 60 items, meaning the time required from both the clinicians and the dyad to complete the assessment is high. Future studies could refine measures by specifying the developmental outcomes assessed by each construct or subscale (e.g., academic, cognitive, behavioural, or socio-emotional development).

Manuals for the identified measures were not often freely available or published. Information about the measure’s psychometric evidence, within accessible manuals, would help to enable clinicians and researchers to be able to make well-informed decisions when choosing assessment measures and prioritise choosing measures that demonstrate good psychometric evidence.

Practical constraints, such as costs, training, manual availability, and required settings or equipment, should also be considered. Additionally, it is also worth considering that parent–infant interactions were often reported to be observed in clinical or laboratory settings; thus, the behaviours rated by clinicians may not be a true reflection of the parent and infant’s typical behaviour in their home environment (116). Further studies could focus more on observations of parent–infant behaviours in the naturalistic home environment.

## Conclusion

Twenty-five parent–infant assessment measures were identified, assessed for risk of bias, and appraised for the quality of their psychometric properties. This review highlights that further research examining the reliability and validity of existing measures is required to advance this field of assessing the parent–infant relationship because few measures could be recommended for clinical and/or research use based on the findings. Clinicians and researchers should be aware of the quality disparities across assessment measures and may need to look beyond local guidelines or clinical recommendations when choosing parent–infant assessment measures.

Although it is reassuring to see a wealth of emerging literature on clinician-rated parent–infant assessment measures, there is a clear need to continue evaluating the existing assessment measures for their reliability and validity to ensure high quality parent–infant assessments, with clinical utility, are completed. More significant efforts should be made to improve the quality of the existing parent–infant assessment measures, as well as increased rigour and transparency in reporting measure development and evaluations, which in turn could serve to enable greater precision, sensitivity and specificity when assessing the parent–infant relationship. Improved detection of any problems or risks within the parent–infant relationship could help to reduce negative consequences for the parents and infants in the future, as well as to facilitate and contribute to the development of interventions within community and clinical PMH services.
