# Supplementary material for: Assessing the parent–infant relationship: a two-stage, COSMIN-informed systematic review evaluating clinician-rated measures
Source: Front Psychiatry. 2025 Apr 28;16:1426198. doi: 10.3389/fpsyt.2025.1426198 (PMC12066551; doi:10.3389/fpsyt.2025.1426198)
Supplement: Supplementary file 1 [file Table1.docx]

Supplementary materials

**Appendix A:** **COSMIN evaluation sheet**

| Category | Criteria | Scoring guidelines |
| --- | --- | --- |
| Content validity: Relevance | Was the content validity established? | V – Content validity index calculated based on the expert opinion  A – Based on expert opinion without an index  D – Unclear/not reported  I – Content validity not established appropriately/adequately  N/A - Not applicable |
|  | Was an appropriate method used to ask professionals whether each item is relevant for the construct of interest?  e.g. evaluated the relevance/appropriateness/suitability and/or acceptability of each item in the target population | V – Clear information given evaluating relevance and appropriate method used  A – Some information given on evaluating the relevance  D – Unclear/not reported  I – Inappropriate method used  N/A - Not applicable |
| Content validity: Comprehensiveness | Was an appropriate method used to ask professionals whether each item is clear for the construct of interest? | V – Clear information given evaluating the comprehensiveness and appropriate method used  A – Some information given on evaluating the comprehensiveness  D – Unclear/not reported  I – Inappropriate method used  N/A - Not applicable |
|  | Does the measure include descriptors of both parent and infant related behaviours to assess? | V - Clear descriptions given  A – Some descriptions given/lack of descriptors acknowledged as a flaw  D – Unclear/not reported  I – Descriptors not appropriate  N/A - Not applicable |
| Content validity: Comprehensibility | Was the comprehensibility evaluated by the end-user? | V – Performed  A – Mentioned but not clearly  D – Unclear/not reported  I – Not evaluated appropriately  N/A - Not applicable |
|  | Was an appropriate method used to assess the comprehensibility – regarding to instructions, items and response options? | V – Appropriate method used  A – Defined during methodology discussion  D – Unclear/not reported  I – method used not appropriate  N/A - Not applicable |
| Structural validity | Was an appropriate method of analysis used to assess the structural validity? | V – An appropriate analysis method used (CTT or IRT)  A – Defined during methodology discussion  D – Unclear/not reported  I – method used not appropriate  N/A - Not applicable |
|  | Was an appropriate exploratory or confirmatory factor analysis chosen? | V – Rational for factor analysis stated, described and justified. Methods and results for checking of the assumptions, method of estimation, goodness-of-fit statistics and cut-off points for good model fit described.  A - Some descriptions of rational/justifications given  D – Unclear/not reported  I – analysis chosen not appropriate  N/A |
|  | Was type of IRT/Rasch model reported? | V – Type of analysis reported as well as method of estimation, methods and results for checking of the assumptions, local dependency, monotonicity, goodness-of-fit statistics, and cut-off points for goodness of item/model fit reported, and all item parameters  A - Limited information given  D – unclear/not reported  I – not assessed appropriately/adequately  N/A |
| Internal consistency | Was the internal consistency calculated and reported? | V – Internal consistency was calculated and reported (Cronbach’s alpha calculated)  A – Only item-total (or inter-items) correlations calculated  D – Unclear/not reported  I – Not assessed appropriately/adequately  N/A - Not applicable |
|  | Were there any other important flaws? | V – no other important methodological flaws  A – other minor methodological flaws  D – Unclear/not reported  I – other important methodological flaws |
| Reliability | Was inter-rater reliability reported? | V – Yes, inter-rater reliability calculated and reported  A – Reasons to assume standard was met  D – Inter-rater reliability not clearly reported and/or unclear  I – No |
|  | Were participants stable in the time between the repeated measurements on the construct to be measured? | V – Yes, evidence provided  A – reasons to assume standard was met  D – unclear  I – No (evidence provided )  N/A – not applicable |
|  | Was intra-rater reliability reported? | V - Yes, Intra-rater reliability calculated and reported and evidence provided  A – Intra-rater reliability assumed to be calculated and standards met  D – Intra-rater not clearly reported/not reported  I – Not appropriate method used to calculate intra-rater reliability  N/A – not applicable |
|  | Was the time interval between the repeated measurements appropriate? | V – Time interval appropriate (at least a month)  D – whether the time interval was appropriate or time interval was not stated/reported  I – Time interval not appropriate  N/A – not applicable |
|  | Were the test conditions similar for the measurements? E.g. type of administration, environment, instructions | V - Test conditions were similar (evidence provided ) using video/image  A – assumable that test conditions were similar  D – unclear if test conditions were similar/not reported  I – test conditions were not similar  N/A – not applicable |
|  | Did the professional(s) administer the measurement without knowledge of scores or values of other repeated measurements in the same participants? | V – Yes (evidence provided)  A – Reasons to assume standard was met  D – Unclear  I – No (evidence provided) |
|  | Did the professionals assign scores or determine values without knowledge of the scores or values of other repeated measurements in the same participants? | V – Yes (evidence provided)  A – Reasons to assume standard was met  D – Unclear  I – No (evidence provided) |
|  | For continuous scores: was an intraclass correlation coefficient (ICC) calculated? | V – ICC calculated; the model or formula was described, and matches study design and the data  A - ICC calculated but model or formula was not described or does not optimally match the study design OR Pearson or Spearman correlation coefficient calculated WITH evidence provided that no systematic difference between measurements has occurred  D – unclear or not reported  I - Pearson or Spearman correlation coefficient calculated WITHOUT evidence provided that no systematic difference between measurements has occurred OR WITH evidence provided that systematic difference between measurements has occurred  N/A |
|  | For ordinal scores: was a (weighted) Kappa calculated? | V – Kappa calculated; the weighting scheme was described, and matches the study design and the data  A - Kappa calculated, but weighting scheme not described or does not optimally match the study design  D - unweighted Kappa calculated instead of weighted Kappa for ordinal scores  I – no kappa/weighted Kappa reported  N/A |
|  | For dichotomous/nominal scores: was Kappa calculated for each category against the other categories combined? | V – Kappa calculated for each category against the other categories combined  N/A |
|  | Were there any other important flaws in the design or statistical methods of the study? | V – No  D – Minor methodological flaws  I - Yes |
| Measurement error | Were sensitivity, specificity and/or accuracy determined? | V – Sensitivity, specificity, and accuracy (% or AUC) calculated for the scale’s score  A – possible to be calculated form the data presented  D – Unclear/not reported  I – sensitivity, specificity and accuracy calculated based on a dichotomous global evaluation based on observer opinion |
|  | Were participants stable in the time between repeated measurements on the construct to be measured? | V – Yes (evidence provided)  A – Reasons to assume standard was met  D – Unclear  I – No (evidence provided)  N/A – Not Applicable |
|  | Were the time intervals between the repeated measurements appropriate? | V – Yes (evidence provided)  A – reasons to assume adequate time interval but more information needed  D – Unclear  I – No (evidence provided)  N/A – Not Applicable |
|  | Were the measurement conditions similar for the repeated measurements? (except for the conditions being evaluated as a source of variation?) | V – Yes (evidence provided)  A – Reasons to assume standard was met OR change was unavoidable  D – Unclear  I – No (evidence provided)  N/A – Not Applicable |
|  | Did the professionals administer the measurement without knowledge of scores or values of other repeated measurements in the same participants? | V – Yes (evidence provided)  A – Reasons to assume standard was met  D – Unclear  I – No (evidence provided) |
|  | Did the professionals assign scores or determine values without knowledge of the scores or values of other repeated measurements in the same participants? | V – Yes (evidence provided)  A – Reasons to assume standard was met  D – Unclear  I – No (evidence provided) |
|  | Were there any other important flaws? | V – No  D – Minor methodological flaws  I – Yes |
|  | For continuous scores: was the Standard Error of Measurement (SEM), Smallest Detectable Change (SDC), Limits of Agreement (LoA) or Coefficiant of Variation (CV) calculated? | V - SEM, SDC, LoA or CV calculated; the model or formula for the SEM/SDC is described; it matches the reviewer constructed research question and the data  A - SEM, SDC, LoA or CV calculated, but the model or formula is not described or does not optimally match the reviewer constructed research question and evidence provided that no systematic difference has occurred  D – SEM consistency, SDC consistency or LoA or CV calculated, without knowledge about systematic difference or with evidence provided that systematic difference has occurred or not reported  I – SEM calculated based on cronbach’s alpha, or using SD from another population  N/A - (dichotomous/nominal/ordinal scores) |
|  | For dichotomous/nominal/ordinal scores: Was the percentage specific (e.g. positive and negative) agreement calculated? | V - % specific agreement calculated  A - % agreement calculated  I – Not reported?  N/A |
| Criterion validity | Was criterion validity reported? | V – Criterion validity was calculated and reported  A – comparison to another measure but further information needed  D – Unclear  I – not reported.  N/A – Not applicable |
|  | Is it clear what the gold standard or other method measures? | V – construct is clear  D – Unable to determine  I – Construct is not clear |
|  | Were the measurement properties of the gold standard or other validated methods adequate? | V – Sufficient evidence of the measurement properties of the gold standard or other method provided (reference included)  A - Partial evidence of the measurement properties of the gold standard or other method provided  D - No information on the measurement properties of the gold standard or other method  I – Evidence for insufficient measurement properties of the gold standard or other method |
|  | Was the statistical method appropriate for the hypotheses to be tested? | V – Statistical method was appropriate (correlations or area under the ROC curve calculated)  A - Assumable that statistical method was appropriate  D – statistical method applied not clearly reported  I - statistical method applied not appropriate |
|  | Were there any other important flaws? | V – no other important methodological flaws  A or D – other minor methodological flaws  I – other important methodological flaws |
| Hypothesis testing for construct validity | Was construct validity reported? | V – construct validity was reported  A – assumable this standard was met but not enough information given  D – Unclear  I – construct validity not met |
|  | Was an adequate description provided of important characteristics of the subgroups? | V – adequate description of the important characteristics of the subgroups (minimum age, sex)  A - adequate description of most of the important characteristics of the subgroups  D – poor or no description of the important characteristics of the subgroups |
|  | Was the statistical method appropriate for the hypotheses to be tested? | V – statistical method was appropriate  A – assumable that the statistical method was appropriate  D – statistical method applied not optimal or not clear  I – statistical method applied not appropriate |
|  | Were there any other important flaws? | V – no other important methodological flaws  A or D – other minor methodological flaws  I – other important methodological flaws |
| Responsiveness | Was responsiveness reported? | V – responsiveness was calculated and reported  A – reasons to assume responsiveness was calculated but not enough information given  D – unclear  I – Not calculated/not reported |
|  | Was an adequate description provided of the assessment measure given? | V – adequate description of the measure and appropriate interval (before-after) considered  A – description of the measure not optimal and/or interval (before-after) not considered  D - poor description of measure and/or interval (before/after)  I – no description of the measure and/or interval (before-after) not appropriate |
|  | Was the statistical method appropriate for the hypotheses to be tested? | V – statistical method was appropriate  A - Assumable that statistical method was appropriate  D – statistical method applied not optimal or not clearly reported  I - statistical method applied not appropriate |
|  | Were then any other important flaws? | V – no other important methodological flaws  A or D – other minor methodological flaws  I – other important methodological flaws |

| **Appendix B: List of excluded measures** | | |
| --- | --- | --- |
| ***Assessments Excluded at Stage 1*** | | |
|  | **Assessment, brief description, authors/developer(s), year and reference** | **Exclusion reason** |
| **1** | **Adult attachment interview**  The Adult Attachment Interview was developed and designed by Main and co-workers to measure individual differences in representations of attachment figures in adults.  **Authors:** George, Kaplan and Main (1996)  **Reference:**  George C., Kaplan N., Main M. (1996). Adult Attachment Interview (3rd ed.). Unpublished manuscript, University of California at Berkeley, USA. | Assessment does not meet the inclusion criteria because it was designed to be completed by the parent/caregiver rather than an observer/clinician. |
| **2** | **Alarm Distress Baby scale** (**ADBB)**  Sustained withdrawal behavior in infancy is an important alarm signal to draw attention to both organic and relationship disorders. ADBB is a withdrawal scale, for infants between 2 and 24 months of age.  **Authors:** Guedeney and Fermanian (2001)  **Reference:**  Guedeney, A., and Fermanian, J. (2001). A validity and reliability study of assessment and screening for sustained withdrawal reaction in infancy: The Alarm Distress Baby Scale. *Infant Mental Health Journal: Official Publication of The World Association for Infant Mental Health*, *22*(5), 559-575. <https://doi.org/10.1002/imhj.1018> | Assessment does not meet inclusion criteria as it primarily measures other constructs than the parent-infant relationship. This measure assesses social withdrawal. |
| **3** | **Assessment of Mother-Child Interaction with the Etch-a-Sketch (AMCIES)**  The mother-infant dyad are recorded during a standardized play situation using an Etch A Sketch, a toy that creates pictures by two buttons, one that draws horizontal lines and one that draws vertical lines. They are instructed that the mother should use one button and the child the other; thus both have to work together to copy the template. Sample used was children aged 6 and 8 years old.  **Authors:** Jaekel et al. (2012)  **Reference:**  Jaekel, J., Wolke, D. and Chernova, J. (2012). Mother and child behaviour in very preterm and term dyads at 6 and 8 years. Developmental medicine and child neurology, 54, 716-23. https://doi.org/10.1111/j.1469- 8749.2012.04323.x. | Assessment does not meet inclusion criteria as it is not suitable for use with children aged 0-2 years old. |
| **4** | **Atypical Maternal Behavior Instrument for Assessment and Classification (AMBIANCE)**  Trained coders write a narrative of the interaction and record the frequency of disrupted behaviors displayed by caregivers according to each observation of caregiver attachment behaviours.  **Developers:**  Bronfman, Madigan, and Lyons-Ruth, (2009–2014)  **Reference:**  Bronfman, E., Madigan, S., and Lyons-Ruth, K. (2009–2014). Disrupted Maternal Behavior Instrument for Assessment and Classification (AMBIANCE): Manual for coding disrupted affective communication (2nd ed.). Unpublished manuscript, Harvard University Medical School. | Assessment does not meet the inclusion criteria because it only assessed either the parent or the infant but not both. This assessment assesses the caregiver, and primarily focuses on attachment. |
| **5** | **Behavioral Observation of the Newborn Educational Trainer for Teaching Newborn Behavior (BONET)**  A learning aid that educates clinicians about newborn behavioral organization, self-regulation skills, and interactive capabilities.  **Developers:** Karl and Keefer (2011)  **Reference:** Karl, D. J. and Keefer, C. H. (2011). Use of the Behavioral Observation of the Newborn Educational Trainer for Teaching Newborn Behavior. *Principles and Practice. 40*(1), 75-83. <https://doi.org/10.1111/j.1552-6909.2010.01202.x> | Assessment does not meet the inclusion criteria because it only assessed either the parent or the infant but not both. Only assesses the infant. |
| **6** | **Behaviour Coding Scheme**  Assesses the caregiver only. Focuses on the following components in assessment: Command, Warning, Ask, Attend, Reinforce  **Authors:** Forehand and McMahon (1981)  **Reference:**  Forehand RL, McMahon RJ. *Helping the noncompliant child: A clinician’s guide to parent training.* New York: Guilford Press; 1981. | Assessment does not meet the inclusion criteria because it only assessed either the parent or the infant but not both. Only assesses the caregiver. |
| **7** | **Carey Questionnaire**  A 'parent report' questionnaire that determines behavioral style in each of the nine categories of temperament in infants, toddlers and children up to 13 years of age.  **Author:** Carey (1970)  **Reference:**  Carey, W. B. (1970). A simplified method for measuring infant temperament. *The Journal of Pediatrics*, *77*(2), 188-194. | Assessment does not meet the inclusion criteria because it was designed to be completed by the parent/caregiver rather than an observer/clinician. |
| **8** | **Categorical System for Micro-Analysis of the Early Mother–Child Interaction**  Unable to locate full texts of original paper and in none in English Language.  **Developers:** Jorg et al. (1994)  **Reference:**  Jo¨rg M, Dinter R, Rose F, Villalba-Yantorno P, Esser G, Schmidt MH, Laucht M: Kategoriensystem zur Mikroanalyse der fru¨hen Mutter-Kind-Interaktı´on. [Categorical system for a micro analysis of the early mother–child interaction]. Z Kinder Jugendpsychiat 22:97–106, 1994. | Assessment does not meet inclusion criteria due to not being available in English language. |
| **9** | **Childhood Experience of Care and Abuse questionnaire (CECA Q)**  A brief self-report questionnaire. This assesses loss of parents, neglect, antipathy from main carers and physical and sexual abuse in carers.  **Developers:** Bifulco, Bernazzani, Moran and Jacobs (2005)  **Reference:**  Bifulco A, Bernazzani O, Moran PM and Jacobs C (2005) Childhood Experience of Care and Abuse Questionnaire (CECA.Q) Validation in a community series. British Journal of Clinical Psychology, 44: 563-581 | Assessment does not meet the inclusion criteria because it was designed to be completed by the parent/caregiver rather than an observer/clinician. |
| **10** | **Child behaviour checklist (CBCL)**  This measure is a component of the Achenbach System of Empirically Based Assessment (ASEBA). The ASEBA is used to detect behavioural and emotional problems in children and adolescents. The CBCL is completed by parents.  **Authors:** Achenback, T. M. and Rescorla, L. A. (2001)  **Reference:**  Achenbach, T.M., and Rescorla, L.A. (2001). Manual for the ASEBA School-Age Forms and Profiles. Burlington, VT: University of Vermont, Research Center for Children, Youth, and Families. | Assessment does not meet the inclusion criteria because it was designed to be completed by the parent/caregiver rather than an observer/clinician. |
| **11** | **Child Behaviour Checklist-Dysregulation Profile (CBCL-DP)**  A Dysregulation Profile score (CBCL-DP) can be computed by summing the *T-*scores from the Anxious/Depressed, Attention Problems, and Aggressive Behavior syndrome scales of the Child Behavior Checklist. The CBCL-DP was originally developed to identify children with [bipolar disorder](https://www.sciencedirect.com/topics/medicine-and-dentistry/bipolar-disorder) ([Althoff, 2010](https://www.sciencedirect.com/science/article/pii/S0165032718317385?via%3Dihub#bib0003), [Biederman et al., 1995](https://www.sciencedirect.com/science/article/pii/S0165032718317385?via%3Dihub#bib0014)).  **Developer:** Bierderman et al. (1995)  **Reference:** Biederman, J., Wozniak, J., Kiely, K., Ablon, S., Faraone, S., Mick, E., Mundy, E., and Kraus, I. (1995). CBCL clinical scales discriminate prepubertal children with structured interview-serived diagnosis of mania from those with ADHD. *Journal of the American Academy of Child and Adolescent Psychiatry, 34* (4), 464-471. <http://doi.org/10.1097/00004583-199504000-00013> (Original CBCL references above; Achenbach and Rescorla, 2001) | Assessment does not meet the inclusion criteria because it only assessed either the parent or the infant but not both. Only assesses the infant. |
| **12** | **Edinburgh postnatal depression scale**  10-item self-report scale to screen for postnatal depression.  **Developers:** Cox, Holden and Sagovsky (1987)  **Reference:**  Cox, J. L., Holden, J. M., and Sagovsky, R. (1987). Detection of postnatal depression. Development of the 10-item Edinburgh Postnatal Depression Scale. *The British journal of psychiatry : the journal of mental science*, *150*, 782–786. <https://doi.org/10.1192/bjp.150.6.782> | Assessment does not meet inclusion criteria as it primarily measures other constructs than the parent-infant relationship. This assessment primarily assesses postpartum depression. |
| **13** | **Erikson Scales**  Assesses the quality of attachment and behaviour problems in preschool children at high risk.  **Authors:** Erickson, Sroufe and Egeland (1985)  **Reference:**  Erickson, M.F., Sroufe, L.A., and Egeland, B. (1985). The relationship between quality of attachment and behavior problems in preschool in a high-risk sample. *Monographs of the Society for Research in Child Development, 50 1-2*, 147-66 . DOI:[10.2307/3333831](https://doi.org/10.2307/3333831) | Assessment does not meet inclusion criteria as it primarily measures other constructs than the parent-infant relationship. This assessment is designed to assess attachment and behaviour problems. |
| **14** | **Frightened/Frightening (FR)**  A coding system for identifying and scoring the intensity of the three primary forms of FR behavior (frightened, threatening, and dissociative).  **Authors:** Hesse and Main (2006)  **Reference:**  Hesse, E. and Main, M. (2006). Frightened, threatening, and dissociative parental behavior in low-risk samples: Description, discussion, and interpretations. Development and psychopathology, 18, 309-43. <https://doi.org/10.1017/S0954579406060172>. | Assessment does not meet the inclusion criteria because it only assessed either the parent or the infant but not both. Only assesses parental behaviour. |
| **15** | **Home Visit Rating Scale (HOVRS)**  These scales comprise an observational measure of the quality of home-visit practices by staff and family engagement.  **Developers:**  Roggman, et al. (2008)  **Reference:**  Roggman, L. A., Cook, G. A., Jump Norman, V. K., Christiansen, K., Boyce, L. K., and Innocenti, M. S. (2008). The Home Visit Rating Scales (HOVRS). In L. A. Roggman, L. K. Boyce, and M. S. Innocenti, Developmental parenting: A guide for early childhood practitioners (pp. 209–217). Baltimore, MD: Brookes. | Assessment does not meet inclusion criteria as it primarily measures other constructs than the parent-infant relationship. This assessment is designed to assess staff conducting home visits. |
| **16** | **How I Feel About My Baby Now Scale (FAB)**  This is a 10-item scale and is intended as a measure of parental attachment and, consequently, can be filled out by the mother or father.  **Developers:**  Leifer (1997)  **Reference:**  Leifer, M. (1997). Psychological changes accompanying pregnancy and motherhood. *Genetic Psychology Monographs, 95*(1), 55–96. | Assessment does not meet the inclusion criteria because it was designed to be completed by the parent/caregiver rather than an observer/clinician. |
| **17** | **Index of Mother-Infant Separation (IMIS)**  This is a 37-item observational measure of the process of mother-infant contact or separation post birth.  **Authors:** Anderson et al. (2004)  **Reference:**  Anderson, G. C., Radjenovic, D., Chiu, S. H., Conlon, M., and Lane, A. E. (2004). Development of an observational instrument to measure mother-infant separation post birth. *Journal of Nursing Measurement*, *12*(3), 215-234. DOI:10.1891/jnum.12.3.215 | Assessment does not meet inclusion criteria as it primarily measures other constructs than the parent-infant relationship. Assessing the parent-infant relationship is a subscale on a longer inventory. |
| **18** | **Infant-Toddler Home Observation for Measurement of the Environment (IT-HOME)**  To measure the quality and quantity of stimulation and support available to child in the home. The focus is on the child in the environment, as well as the child as a recipient of inputs from objects, events, and interactions occurring around him/her. A systematic assessment of the caring environment in which the child is reared.  **Developers:** Caldwell and Bradley (2001)  **Reference:** Caldwell, B. M., and Bradley, R. H. (2001). HOME inventory and administration manual. (3rd ed.). University of Arkansas for Medical Sciences and University of Arkansas at Little Rock. | Assessment does not meet inclusion criteria as it primarily measures other constructs than the parent-infant relationship. Assessing the parent-infant relationship is a subscale on a longer inventory. |
| **19** | **Korean Paternal-Fetal Attachment scale**  Developed to measure the level of attachment between the father and the expected baby, and to examine its validity and reliability.  **Developers:** Noh and Yeom (2017)  **Reference:** Noh, N. I., and Yeom, H. A. (2017). Development of the Korean paternal-fetal attachment scale (K-PAFAS). *Asian Nursing Research*, *11*(2), 98-106. doi: 10.1016/j.anr.2017.05.001. | Assessment does not meet the inclusion criteria because it was designed to be completed by the parent/caregiver rather than an observer/clinician. |
| **20** | **Maternal ambivalence scale (MAS)**  The MAS includes items that evaluate each of three elements of maternal ambivalence: doubts, conviction, and coping.  **Developers:**  Martín-Sánchez et al. (2022)  **Reference:**  Martín-Sánchez, M.B., Martínez-Borba, V., Catalá, P. Osma, J., Penacoba-Puente, C and Suso-Ribera, C*.* (2022). Development and psychometric properties of the maternal ambivalence scale in spanish women. *BMC Pregnancy Childbirth* 22, 625 <https://doi.org/10.1186/s12884-022-04956-w> | Assessment does not meet inclusion criteria as it primarily measures other constructs than the parent-infant relationship. Maternal attachment only. |
| **21** | **Maternal Antenatal Attachment Scale (MAAS)**  The MAAS is used to assess the quality of the mother-fetus relationship in terms of overt (i.e., conscious) thoughts, feelings, attitudes and behaviors of the mother towards the fetus. It is self-report by the mother.  **Developer:** Condon (1993)  **Reference:**  Condon, J. T. (1993). The assessment of antenatal emotional attachment: Development of a questionnaire instrument. *British Journal of Medical Psychology, 66*, 167–183. | Assessment does not meet the inclusion criteria because it was designed to be completed by the parent/caregiver rather than an observer/clinician. |
| **22** | **Maternal attachment inventory (MAI-26)**  The MAI was developed and tested to provide a practical measure of maternal affectionate attachment. Completed by the mother.  **Developer:**  Müller (1994)  **Reference:**  Müller, M. E. (1994). A questionnaire to measure Mother-to-Infant attachment. *Journal of Nursing Measurement, 2*(2), 129–141. | Assessment does not meet the inclusion criteria because it was designed to be completed by the parent/caregiver rather than an observer/clinician. |
| **23** | **Maternal Behavior Coding System (MBCS)**  The tool consists of main categories and subcategories of maternal caregiving behavior that are structured into two main domains–typical caregiving behavior and atypical caregiving behavior.  **Developer:** Warnock et al. (2009)  **Reference:**  Warnock, F. F., Bakeman, R., Shearer, K., Misri, S., and Oberlander, T. (2009). Caregiving behavior and interactions of prenatally depressed mothers (antidepressant‐treated and non‐antidepressant‐treated) during newborn acute pain. *Infant Mental Health Journal: Official Publication of The World Association for Infant Mental Health*, *30*(4), 384-406. | Assessment does not meet the inclusion criteria because it only assessed either the parent or the infant but not both. This assessment only assesses the mother. |
| **24** | **Maternal Behavior Q-Sort**  This is an observational coding instrument that was developed with specific reference to the original Ainsworth sensitivity scale, which defines sensitivity as a caregiver’s ability to perceive, accurately interpret and adequately respond to the needs of the infant.  **Authors:** Pederson et al. (1990)  **Reference:**  Pederson, D. R., Moran, G., Sitko, C., Campbell, K., Ghesquire, K., and Acton, H. (1990). Maternal sensitivity and the security of infant‐mother attachment: AQ‐sort study. *Child development*, *61*(6), 1974-1983. <https://doi.org/10.1111/j.1467-8624.1990.tb03579.x> | Assessment does not meet the inclusion criteria because it only assessed either the parent or the infant but not both. This assessment only assesses the mother. |
| **25** | **Maternal Behaviour Q-Sort Mini**  The Mini-MBQS was designed to be less time consuming to code than the long-form outlined above.  **Author:** Moran (2009)  **Reference:** Moran, G. (2009). Mini-MBQS-V Revised Mini-MBQS 25 item for video coding. Retrieved from <http://works.bepress.com/gregmoran/49/> | Assessment does not meet the inclusion criteria because it only assessed either the parent or the infant but not both. This assessment only assesses the mother. |
| **26** | **Maternal Behaviour Rating Scale (MBRS)**  The MBRS is a series of 18 items compiled from various global maternal rating scales reported in the child development literature.  **Authors:** Mahoney, Finger and Powell (1985)  **Reference:** Mahoney G., Finger, I., and Powell, A. (1985). The relationship of maternal behavioral style to the developmental status of organically impaired mentally retarded infants. American Journal of Mental Deficiency, 90 (3), 296-30 | Assessment does not meet the inclusion criteria because it only assessed either the parent or the infant but not both. This assessment only assesses the mother. |
| **27** | **Maternal Fetal Attachment Scale**  A 24-item scale with five subscales developed to measure the construct of maternal-fetal attachment during pregnancy.  **Developers:** Cranley (1981)  **Reference:** Cranley, M. S. (1981). *Maternal Fetal Attachment Scale (MFAS)* [Database record]. APA PsycTests.  [**https://doi.org/10.1037/t06786-000**](https://psycnet.apa.org/doi/10.1037/t06786-000) | Assessment does not meet the inclusion criteria because it was designed to be completed by the parent/caregiver rather than an observer/clinician. |
| **28** | **Maternal Infant Responsive Instrument (MIRI)**  The MIRI was mainly developed to estimate the ability of the mother to recognise their responsiveness to their babies. The primary belief is that mothers can acknowledge their success in understanding the signals of their infants, giving the right responses and when their babies respond to the attempts. The MIRI is a practical self-report tool that can be completed anytime anywhere within a few minutes.  **Developers:** Drake et al. (2007)  **Reference:** Drake, E. E., Humenick, S. S., Amankwaa, L., Younger, J., and Roux, G. (2007). Predictors of maternal responsiveness. *Journal of Nursing Scholarship*, *39*(2), 119-125. <https://doi.org/10.1111/j.1547-5069.2007.00156.x> | Assessment does not meet the inclusion criteria because it was designed to be completed by the parent/caregiver rather than an observer/clinician. |
| **29** | **Maternal infant synchrony scale**  This is an observational assessment tool designed to assess synchrony during feeding interactions by investigating a concept similar to synchrony: engagement.  **Developer:** Reyna et al. (2012)  **Reference:**  Reyna, B. A., Brown, L. F., Pickler, R. H., Myers, B. J., and Younger, J. B. (2012). Mother–infant synchrony during infant feeding. *Infant Behavior and Development*, *35*(4), 669-677. <https://doi.org/10.1016/j.infbeh.2012.06.003> | Assessment does not meet inclusion criteria as it primarily measures other constructs than the parent-infant relationship. Infant synchrony/engagement is the main construct being measured. |
| **30** | **Maternal postnatal attachment scale (MPAS)**  This measure assesses mother-to-child attachment using a 19 item self-report questionnaire.  **Developer:** Condon and Corkindale (1998)  **Reference:**  Condon, J. T., and Corkindale, C. J. (1998). The assessment of parent-to-infant attachment: Development of a self-report questionnaire instrument. *Journal of Reproductive and Infant Psychology, 16*(1), 57–76. DOI:10.1080/02646839808404558 | Assessment does not meet the inclusion criteria because it was designed to be completed by the parent/caregiver rather than an observer/clinician. |
| **31** | **Mother-Infant Togetherness Scale**  A survey to assess mother-infant “togetherness”. Completed by the mother.  **Authors:** Lawrence (2012)  **References:**  Lawrence, C. (2012). Development And Evaluation Of An Instrument To Measure Mother-infant Togetherness After Childbirth. | Assessment does not meet the inclusion criteria because it was designed to be completed by the parent/caregiver rather than an observer/clinician. |
| **32** | **Mother and baby interaction scale (MABISC)**  The scale was developed in a Parent and Baby Unit in the UK, based on clinical experience with interviews with depressed mothers. The questions reflected the mothers’ most commonly reported reactions to their infants in these interviews.  **Authors:**  Hackney et al. (1996)  **Reference:** Hackney, M., Braithwaite, S., Radcliff, G. (1996). Postnatal depression: the development of a self-report scale. *Health Visitor, 169,* 103-104. | Assessment does not meet the inclusion criteria because it was designed to be completed by the parent/caregiver rather than an observer/clinician. |
| **33** | **Mother infant attachment scale (MIAS)**  A 15 item mother-infant attachment scale, developed to be completed by mothers.  **Authors:** Bhakoo et al. (1994)  **Reference:**  Bhakoo, O. N., Pershad, D., Mahajan, R., and Gambhir, S. K. (1994). Development of mo- ther-infant attachment scale. *Indian Pediatrics, 31*, 1477–1482. | Assessment does not meet the inclusion criteria because it was designed to be completed by the parent/caregiver rather than an observer/clinician. |
| **34** | **Mother-infant psychoanalysis (MIP)**  MIP is based on psychoanalytic theory applied to young children. Sessions occur with infant and mother together.  **Authors:** Norman (2001)  **Reference:**  Norman, J. (2001). The psychoanalyst and the baby: A new look at work with infants. *The International Journal of Psychoanalysis*, *82*(1), 83-100. <https://doi.org/10.1516/MFNV-9KKF-4AJT-8RC1> | Assessment does not meet inclusion criteria due to being an intervention. |
| **35** | **Mother-to-infant bonding scale (MIBS)**  This measure was developed as a simple 8-item self-rating mother-to-infant bonding questionnaire designed to measure the feelings of a mother towards her new baby.  **Authors:** Taylor et al. (2005)  **Reference:**  Taylor, A., Atkins, R., Kumar, R., Adams, D., and Glover, V. (2005). A new mother-to-infant bonding scale: Links with early maternal mood. *Archives of Women’s Mental Health, 8*(1), 45–51. <https://doi.org/10.1007/s00737-005-0074-z> | Assessment does not meet the inclusion criteria because it was designed to be completed by the parent/caregiver rather than an observer/clinician. |
| **36** | **Mother-to-infant relations and feelings scale (MIRFS)**  This scale was developed to assess mothers’ self-reported relation to and feelings for her baby. The scale was developed to evaluate the effect of a process oriented training program aiming to improve professional support from antenatal midwives and postnatal nurses  **Authors:** Thorstensson et al. (2012)  **Reference:**  Thorstensson, S., Hertfelt Wahn, E., Ekström, A., and Langius-Eklöf, A. (2012). Evaluation of the Mother-to-Infant relation and feeling scale: Interviews with first-time mothers’ for feelings and relation to their baby three days after birth. *International Journal of Nursing and Midwifery*, *4*(1), 8-15. DOI: 10.5897/IJNM11.041 | Assessment does not meet the inclusion criteria because it was designed to be completed by the parent/caregiver rather than an observer/clinician. |
| **37** | **Mother’s object relations scales-short form (MORS-SF)**  A 14-item questionnaire, MORS-SF, was developed to assess mothers’ representations of their infants.  **Authors:** Oates et al. (2018)  **Reference:** Oates, J., Gervai, J., Danis, I., Lakatos, K., and Davies, J. (2018). Validation of the Mothers’ object relations scales short-form (MORS-SF). *Journal of Prenatal and Perinatal Psychology and Health*, *33*(1), 38-50. | Assessment does not meet the inclusion criteria because it was designed to be completed by the parent/caregiver rather than an observer/clinician. |
| **38** | **Mutual regulation model**  In this model infants play a major agentic role in regulating the interaction. They invite fitted, regulatory scaffolding, with meanings conveyed through eye contact, facial expressions, and emotive expressions such as crying or laughing. Caregivers vary in the degree to which they apprehend and learn their infant’s messages and thus vary in how much they help (or hinder) the infant’s regulation.  **Authors:** Gianino and Tronick (1988)  **Reference:** Gianino, A., and Tronick, E. (1988). The mutual regulation model: The infant’s self and interactive regulation and coping and defense capacities. In T. Field, P. McCabe, and N. Schneiderman (Eds.), Stress and coping (pp. 47-68). Mahwah, NJ: Erlbaum. | Assessment does not meet inclusion criteria as it primarily measures other constructs than the parent-infant relationship. Assesses primarily infant emotional regulation. |
| **39** | **Neonatal Behavioral Assessment Scale (NBAS)**  This scale is a comprehensive examination of newborn behavior. It is best described as a neurobehavioral assessment scale, designed to describe the newborn’s responses to his/her new extrauterine environment.  **Authors:** Brazelton (1973, 1984); Brazelton and Nugent (1995)  **Reference:**  Brazelton, T. B., and Nugent, J. K. (1995). *Neonatal behavioral assessment scale* (No. 137). Cambridge University Press. | Assessment does not meet inclusion criteria as it primarily measures other constructs than the parent-infant relationship. This assessment only assess infant behaviour. |
| **40** | **Neonatal Imitation, Gaze Aversion, and Mother-Infant Interaction**  Gaze aversion, tongue protrusion and imitation observed and assessed in order to determine infant social interaction.  **Authors:** Heimann (1989)  **Reference:** Heimann, M. (1989). Neonatal imitation, gaze aversion, and mother-infant interaction. *Infant Behavior and Development, 12*(4), 495–505. [https://doi.org/10.1016/0163-6383(89)90029-5](https://psycnet.apa.org/doi/10.1016/0163-6383(89)90029-5) | Assessment does not meet inclusion criteria as it primarily measures other constructs than the parent-infant relationship. Assessing the parent-infant relationship is a subscale on a longer inventory. |
| **41** | **Neonatal Intensive Care Unit Network Neurobehavioral Scale (NNNS)**  The NNNS offers nurses in clinical practice and research a comprehensive neurobehavioral assessment especially suited to high-risk and premature infants.  **Authors:** Lester and Tronick (2004)  **Reference:** Lester, B. M., and Tronick, E. Z. (2004). The neonatal intensive care unit network neurobehavioral scale (NNNS). *Pediatrics (Evanston)*, *113*(3). | Assessment does not meet the inclusion criteria because it only assessed either the parent or the infant but not both. Only assesses the infant. |
| **42** | **Newborn Behavioural Observation (NBO)**  The NBO is a tool that helps demonstrate the abilities of new-born babies in the first 3 months of life and how each individual baby is unique. The tool highlights to parents and professionals how the baby is using their behaviour to communicate to those around them. The NBO sessions are based on a strength-based approach.  **Authors:** Nugent, Keefer, Minear, Johnson and Blanchard (2007)  **Reference:**  Nugent, J. K., Keefer, C. H., Minear, S., Johnson, L. C., and Blanchard, Y. (2007). The newborn behavioral observations (NBO) system handbook. *Paul H Brookes Publishing, Baltimore, MD, USA*. | Assessment does not meet inclusion criteria due to being an intervention. |
| **43** | **NIMHANS Maternal Behaviour Scale (NIMBUS)**  The NIMHANS Maternal Behaviour Scale (NIMBUS) was developed to assess mother infant interaction in mothers with severe mental illness.  **Authors:** Ganjekar et al. (2020)  **Reference:** Ganjekar, S., Prakash, A., Thippeswamy, H., Desai, G., and Chandra, P. S. (2020). The NIMHANS (National Institute of Mental Health and Neuro Sciences) Maternal Behaviour Scale (NIMBUS): Development and validation of a scale for assessment of maternal behaviour among mothers with postpartum severe mental illness in low resource settings. *Asian journal of psychiatry*, *47*, 101872. <https://doi.org/10.1016/j.ajp.2019.101872> | Assessment does not meet the inclusion criteria because it only assessed either the parent or the infant but not both. Only assesses the mother. |
| **44** | **Observational assessment of mother-baby interaction**  This instrument can be used to evaluate the interactions between newborns and mothers. Contact is assessed by that conducted with blankets and excessive clothing – assesses effect of skin-to-skin contact primarily.  **Authors:** Dumas et al. (2013)  **Reference:**  Dumas, L., Lepage, M., Bystrova, K., Matthiesen, A. S., Welles-Nyström, B., and Widstroem, A. M. (2013). Influence of skin-to-skin contact and rooming-in on early mother–infant interaction: a randomized controlled trial. *Clinical nursing research*, *22*(3), 310-336. <https://doi.org/10.1177/1054773812468316> | Assessment does not meet inclusion criteria as it primarily measures other constructs than the parent-infant relationship. This measure primarily assesses the effect of skin-to-skin contact. |
| **45** | **Observation of routine screen form**  An intervention aimed at enhancing engagement in the ultrasound examination and thereby increasing feelings of connection to the fetus and investment in a healthy pregnancy.  **Authors:** Boukydis et al. (2006)  **Reference:** Boukydis, C. Z., Treadwell, M. C., Delaney-Black, V., Boyes, K., King, M., Robinson, T., and Sokol, R. (2006). Women's responses to ultrasound examinations during routine screens in an obstetric clinic. *Journal of Ultrasound in Medicine*, *25*(6), 721-728. <https://doi.org/10.7863/jum.2006.25.6.721> | Assessment does not meet inclusion criteria due to being an intervention. |
| **46** | **Paternal antenatal attachment scale (PAAS)**  A 16-item paternal questionnaire designed to assess the antenatal attachment construct.  **Authors:** Condon (1993)  **Reference:** Condon, J. T. (1993). The assessment of antenatal emotional attachment: Development of a questionnaire instrument. *British Journal of Medical Psychology, 66*, 167–183. DOI: [10.1111/j.2044-8341.1993.tb01739.x](https://doi.org/10.1111/j.2044-8341.1993.tb01739.x) | Assessment does not meet the inclusion criteria because it was designed to be completed by the parent/caregiver rather than an observer/clinician. |
| **47** | **Paternal postnatal attachment scale (PPAS)**  A tool designed to measure the construct of father-to-infant attachment using a 19-item self-report questionnaire.  **Author:** Condon, Corkindale and Boyce (2008)  **Reference:** Condon, J. T., Corkindale, C. J., and Boyce, P. (2008). Assessment of postnatal paternal–infant attachment: Development of a questionnaire instrument. *Journal of Reproductive and Infant Psychology, 26*(3), 195–210. [https://doi.org/10.1080/02646830701691335](https://psycnet.apa.org/doi/10.1080/02646830701691335) | Assessment does not meet the inclusion criteria because it was designed to be completed by the parent/caregiver rather than an observer/clinician. |
| **48** | **Paternal-fetal attachment scale (PFAS)**  A 63-item self-report questionnaire utilizing the modified Maternal-Fetal Attachment Scale (PFA) (Cranley, 1981),  **Developer:** Weaver and Cranley (1983)  **Reference:** Weaver, R. H., and Cranley, M. S. (1983). An exploration of paternal-fetal attachment behavior. *Nursing Research*, *32*(2), 68-72. | Assessment does not meet the inclusion criteria because it was designed to be completed by the parent/caregiver rather than an observer/clinician. |
| **49** | **Perceived maternal parenting self-efficacy scale (PMP S-E)**  This scale is designed to measure the views that the mothers themselves have about their self-efficacy in caring for a baby. Parent-report measure.  **Authors:** Aliabadi et al. (2013)  **Reference:**  Aliabadi F, Borimnejad L, Kamali M, Rassafiani M, Nazi S. (2013). Perceived Perceived Maternal Parenting Self-Efficacy (PMP SE) tool: translation and face validation with Iranian mothers of hospitalized preterm neonates. *Iranian Rehabilitation Journal*, *11*(1), 7-10. | Assessment does not meet the inclusion criteria because it was designed to be completed by the parent/caregiver rather than an observer/clinician. |
| **50** | **Perinatal Illness perceptions scale (PIPS)**  The PIPS is described as the first robust psychometric measure, which can be used to in the assessment of practitioner knowledge of the causes and consequences of perinatal mental health.  **Authors:** Jomeen, Martin and Jarrett (2019)  **Reference:**  Jomeen, J., Martin, C.R. and Jarrett, P.M. (2019), "Psychometric evaluation of the Perinatal Illness Perceptions Scale (PIPS)", *The Journal of Mental Health Training, Education and Practice*, Vol. 14 No. 2, pp. 74-85. <https://doi.org/10.1108/JMHTEP-09-2018-0054> | Assessment does not meet inclusion criteria as it primarily measures other constructs than the parent-infant relationship. This assessment primarily assesses illness perceptions. |
| **51** | **Perinatal Infant Care Social Support Questionnaire (PICSS)**  A reliable and valid instrument to measure social support for new mothers in the perinatal period.  **Authors:** Leahy-Warren, Mulcahy and Lehane (2019)  **Reference:**  Leahy-Warren, P., Mulcahy, H., and Lehane, E. (2019). The development and psychometric testing of the Perinatal Infant Care Social Support (PICSS) instrument. *Journal of psychosomatic research*, *126*, 109813. <https://doi.org/10.1016/j.jpsychores.2019.109813> | Assessment does not meet the inclusion criteria because it was designed to be completed by the parent/caregiver rather than an observer/clinician. |
| **52** | **Postpartum bonding questionnaire (PBQ)**  This self-report measure consists of 25 items, with four factors, designed to assess and identify any difficulties between the caregiver-infant relationship.  **Authors:** Brockington, Fraser and Wilson (2006)  **Reference:**  Brockington, I. F., Fraser, C., and Wilson, D. (2006). The postpartum bonding questionnaire: a validation. *Archives of women's mental health*, *9*, 233-242. <https://doi.org/10.1007/s00737-006-0132-1> | Assessment does not meet the inclusion criteria because it was designed to be completed by the parent/caregiver rather than an observer/clinician. |
| **53** | **Pre- and the Postnatal Bonding Scale**  A user-friendly self-report questionnaire was developed to assess maternal bonding during pregnancy and postpartum.  **Authors:** Cuijlits et al. (2016)  **Reference:**  Cuijlits, I., van de Wetering, A. P., Potharst, E. S., Truijens, S. E. M., van Baar, A. L., and Pop, V. J. M. (2016). Development of a Pre- and Postnaal Bonding Scale (PPBS). *Journal of Psychology and Psychotherapy, 6*(5), 1000282. DOI:[10.4172/2161-0487.1000282](http://dx.doi.org/10.4172/2161-0487.1000282) | Assessment does not meet the inclusion criteria because it was designed to be completed by the parent/caregiver rather than an observer/clinician. |
| **54** | **Prenatal Attachment Inventory (PAI)**  This self-report scale is designed to assess “the unique‚ affectionate bond that develops between a woman and her fetus”  **Authors:**  Müller (1993)  **Reference:**  Müller, M. E. (1993). Development of the prenatal attachment inventory. *Western Journal of Nursing Research, 15*(2), 199–215. doi: 10.1177/019394599301500205. | Assessment does not meet the inclusion criteria because it was designed to be completed by the parent/caregiver rather than an observer/clinician. |
| **55** | **Preterm Infant Coding System for Maternal Sensitivity (PRICOSMAS)**  The PRICOSMAS assesses Maternal sensitivity (MS), the ability to perceive and synchronously respond to the social signals.  **Authors:** Boissel et al. (2022)  **Reference:** Boissel, L., Pinchaux, E., Guilé, M., Corde, P., Crovetto, C., Diouf, M., Mariana, C., Meynier, J., Picard, C., Scoury, D., Cohen, D., Benarous, X., Viaux-Savelon, S., and Guilé, J. M. (2022). Development and reliability of the coding system evaluating maternal sensitivity to social interactions with 34- to 36-week postmenstrual age preterm infants. *Frontiers in psychiatry*, *13*, 938482. <https://doi.org/10.3389/fpsyt.2022.938482> | Assessment does not meet inclusion criteria as it primarily measures other constructs than the parent-infant relationship. This assessment primarily assesses maternal sensitivity. |
| **56** | **Scale of Observation of the Mother-Baby Bond Interned in the NICU**  This scale was developed to measure parental stress related to the physical and psychosocial environment measured within the NICU.  **Developer:** Santos (2008)  **Reference:**  Santos, M. S. (2008). Construcción de una escala de observación del vínculo madre-bebé internado en UCIN: Resultados preliminares de la aplicación del protocolo de observación. *Anuario de investigaciones*, *15*, 0-0. | Assessment does not meet inclusion criteria as it primarily measures other constructs than the parent-infant relationship. This assessment primarily assesses parental stress in the NICU. |
| **57** | **Short Postpartum Bonding Questionnaire - S-PBQ**  Based on the original 25-item *Postpartum Bonding Questionnaire*  **Authors:** Kinsey et al. (2014)  **Reference:** Kinsey, C. B., Baptiste-Roberts, K., Zhu, J., and Kjerulff, K. H. (2014). Birth-related, psychosocial, and emotional correlates of positive maternal-infant bonding in a cohort of first-time mothers. *Midwifery, 30*, e188–e194. | Assessment does not meet the inclusion criteria because it was designed to be completed by the parent/caregiver rather than an observer/clinician. |
| **58** | **Videofeedback Intervention to promote Positive Parenting and sensitive discipline (VIPP-SD)**  VIPP is a way of working with families that focuses on attachment and the parent/carer-child relationship. It helps parents/carers to care for their child by building a strong relationship with them, and seeing the world through their eyes.  **Authors:** Juffer, Bakermans-Kranenburg and Van Ijzendoorn (2018)  **Reference:** Juffer, F., Bakermans-Kranenburg, M. J., and Van Ijzendoorn, M. H. (2018). Video-feedback intervention to promote positive parenting and sensitive discipline. *Handbook of attachment-based interventions. New York: Guilford*. | Assessment does not meet inclusion criteria due to being an intervention. |
| **59** | **Video Interactive Guidance (VIG)**  VIG is an intervention that promotes secure attachment and optimal development for children, parents and professionals through embodiment of its theoretical framework, values and beliefs and the Principles of Attuned Interaction and Guidance (PAIG).  **Authors:**  Kennedy and Underdown (2018)  **Reference:**  Kennedy, H., and Underdown, A. (2018). Video interaction guidance: Promoting secure attachment and optimal development for children, parents and professionals. In P. Leach (Ed.), *Transforming infant wellbeing: Research, policy and practice for the first 1001 critical days* (pp. 224–237). Routledge/Taylor and Francis Group. [https://doi.org/10.4324/9781315452890-23](https://psycnet.apa.org/doi/10.4324/9781315452890-23) | Assessment does not meet inclusion criteria due to being an intervention. |
| **60** | **Working model of the child interview**  The Working Model of the Child Interview is a structured interview to assess parents' internal representations or working models of their relationship to a particular child.  **Authors:** Zeanah and Benoit (1995)  **Reference:** Zeanah, C. H., and Benoit, D. (1995). *Working Model of the Child Interview (WMCI)* [Database record]. APA PsycTests. <https://doi.org/10.1037/t47439-000> | Assessment does not meet the inclusion criteria because it was designed to be completed by the parent/caregiver rather than an observer/clinician. |
| ***Assessments Excluded at Stage 2*** | | |
|  | **Assessment, brief description, authors/developer(s), year** | **Exclusion reason** |
| **1** | **Behavioural Dialogues**  Describes a method for objectively assessing the style of mother-infant interaction without regard to the specific content of that interaction.  **Authors: Bakeman and Brown (1977)**  **Reference:**  Bakeman, R., and Brown, J. V. (1977). Behavioral Dialogues: An Approach to the Assessment of Mother-Infant Interaction. *Child Development*, *48*(1), 195–203. <https://doi.org/10.2307/1128898> | No eligible reports found. |
| **2** | **Bobigny grid: Grielle Recherche Action Formation (RAF)**  Grid for assessment of basic mother-infant interactions, designed as part of a research-action-training program in France.  **Authors: Bur, Golzan and Lamour (1989)**  **Reference:** Unknown | No eligible reports found in English language. |
| **3** | **Child and Adult Relationship Observation (CARO)**  A tool used to identify positive interactions between parent and child.  **Authors:** Drummond (2020)  **Reference:**  Drummond, J. (2020). *The concurrent validity of the Child and Adult Relationship Observational tool (CARO) for mothers with additional health and social care needs and Clinical Research Portfolio.* D Clin Psy thesis, University of Glasgow | No eligible reports found. |
| **4** | **Global Ratings Scales (GRS)**  Video-based assessments of the quality of mother-infant engagement.  **Authors:**  Murray et al. (1996)  **Reference:**  Murray, L., Fiori-Cowley, A., Hooper, R., Murray, L., Fiori-Cowley, A., Hooper, R., et al (1996b) The impact of postnatal depression and associated adversity on early mother-infant interactions and later infant outcome. *Child Development, 67,* 2512-2526. | Unable to access full-text of eligible reports. |
| **5** | **Greenspan-Lieberman Observational Scale – Revised (GLOS-R)**  Used to investigate the mother-infant relationship.  **Authors:** Greenspan and Lieberman (1989)  **Reference:**  Greenspan, S. I., and Lieberman, A. F. (1989). Infants, mothers, and their interaction: A quantitative clinical approach to developmental assessment. *In S. I. Greenspan and G. H. Pollock (Eds.), The course of life, Vol 1:* Infancy (pp. 503–560). Madison, CT: International Universities Press. | No eligible reports found. |
| **6** | **The Functional Emotional Assessment Scale (FEAS)**  Designed to measure (1) the full range of emotional functioning in infants, young children and their caregivers; (2) naturally occurring emotional interactions between infants, children, and caregivers in a variety of settings, including home and school; and (3) both easily observable emotional behaviours and the subtle, difficult-to-measure, deeper levels of emotional functioning.  **Authors:** Greenspan, S. I., DeGangi, G., and Wieder, S. (2001)  **Reference:**  Greenspan, S. I., DeGangi, G., and Wieder, S. (2001). The Functional Emotional Assessment Scale (FEAS) for infancy and early childhood: Clinical and research applications. Bethesda, MD: Interdisciplinary Council on Developmental and Learning Disorders. | No eligible reports found - book chapter so not published in a peer-reviewed journal. |
| **7** | **Maternal Sensitivity Scale**  Measures maternal sensitivity.  **Authors:** Stiles (2004)  **Reference:**  Stiles A. S. (2004). Measuring maternal sensitivity in teen mothers: reliability and feasibility of two instruments. *Journal of nursing measurement*, *12*(3), 195–214. <https://doi.org/10.1891/jnum.12.3.195> | Unable to access full-text of eligible reports and evaluation/description of measure not stated as an aim of paper. |
| **8** | **Mother-Infant Communication Screening (MICS)**  A screening tool to indicate if a mother-infant dyad may be at risk for a threatening disorder of mother-infant communication.  **Author: Raack (1989)**  **Reference:** Raack, C. B. (1989). Mother/Infant Communication Screening (MICS). Roselle, IL: Community Therapy Services. | Unable to access full-text of eligible reports. |
| **9** | **Mother-Infant Play Interaction Scale (MIPIS)**  No description found.  **Author:** Walker and Thompson (1982)  **Reference:**  Walker, L. O., and Thompson, E. T. (1982). Mother–Infant Play Interaction Scale. In S. S. Humenick (Ed.), Analysis of current assessment strategies in the health care of young children and childbearing families (pp. 191–201). East Norwalk, CT: Appleton-Century-Crofts. | No eligible reports found. book chapter so not published in a peer-reviewed journal. |
| **10** | **National Institute of Child Health and Human Development (NICHD)**  How different childcare arrangements related to measurements of the children’s health.  **Authors:** NICHD Early Child Care Research Network (1997)  **Reference:** NICHD Early Child Care Research Network. (1997). The effects of infant child care on infant-mother attachment security: Results of the NICHD study of early child care. *Child development*, 860-879. | No eligible reports found. |
| **11** | **Nursing Child Assessment Feeding Scales (NCAFS)**  Assesses mother-child interaction in the context of feeding/eating.  **Authors:** Sumner and Spietz (1994)  **Reference:**  Sumner, G., and Spietz, A. (1994). NCAST Caregiver/ Parent-Infant Interaction feeding manual. Seattle, WA: NCAST Publications. | Unable to access full-text of eligible reports. |
| **12** | **Revised Relational Coding System (RCS)**  A competency coding system to measure clinician efficacy.  **Authors:** Fogel et al. (2003)  **Reference:**  Fogel, A., De Koyer, I., Secrist, C., Sipherd, A., Hafen, T., and Fricke, M. (2003). The Revised Relational Coding System. Unpublished manual, Department of Psychology, University of Utah, Salt Lake City, UT. | No eligible reports found. |
| **13** | **The Determinants of Parenting: A Process Model**  A process model of competent parental functioning is offered on the basis of analysis.  **Developer:** Belsky (1984)  **Reference:**  Belsky, J., Taylor, D. G., and Rovine, M. (1984). The Pennsylvania Infant and Family Development Project, II: The development of reciprocal interaction in the mother–infant dyad. Child Development, 55(3), 706– 717. doi:10.1111/1467-8624.ep12422849 | Not an eligible assessment measure (a process model) |
| **14** | **The Dyadic Parent-Child Interaction Coding System-II (DPICS-II)**  A single, adaptable system for recording important parent and child behaviours that maintain particular parent-child interaction patterns associated with ineffective parenting styles and disruptive child behaviour – manual not explicitly evaluating validation or development itself, a review of other studies.  **Developers:** Eyberg and Robinson (1983)  **Reference:**  Eyberg, S. M., and Robinson, E. A. (1983). Dyadic Parent-Child Interaction Coding System (DPICS): A manual. Psychological Documents, 13(2), 24. | No eligible reports found – all validation studies located were dissertation abstracts. |
| **15** | **The Mellow Parenting programme**  An intensive parenting programme for vulnerable families with younger children.  **Authors:** Puckering et al. (1996)  **Reference:**  Puckering, C., Evans, J., Maddox, H., Mills, M., & Cox, A.D. (1996). Taking control: A single case study of mellow parenting. *Clinical Child Psychology and Psychiatry, 1,*  539-550. | No eligible reports found – theses/no information on development/validation of measure/full-text reports not accessible. |
| **16** | **Two bags task**  A standardised, semi-structured observational measures (not suitable for infants under the age of two).  **Developers:** Early Childhood Longitudinal Studies Program (ECLS)  **Reference:** <https://nces.ed.gov/pubs2007/2007084_C6.pdf> | No eligible reports found. |

| **Interpretation Key – Stage 1** |
| --- |
| Assessment does not meet the inclusion criteria because it was designed to be completed by the parent/caregiver rather than an observer/clinician. |
| Assessment does not meet inclusion criteria as it primarily measures other constructs than the parent-infant relationship. |
| Assessment does not meet inclusion criteria as it is not suitable for use with children aged 0-2 years old. |
| Assessment does not meet the inclusion criteria because it only assessed either the parent or the infant but not both. |
| Assessment does not meet inclusion criteria due to not being available in English language. |
| Assessment does not meet inclusion criteria due to being an intervention. |

| **Interpretation Key – Stage 2** |
| --- |
| No eligible reports found. |
| Not an eligible assessment measure (a process model) |
| Unable to access full-text of eligible reports. |
| No eligible reports found in English language. |
